# Metal-Driven Biochemical and Physiological Stress in Fish Inhabiting an Urban Coastal Lagoon: Evidence from an Integrated Multibiomarker Approach

**DOI:** 10.1007/s12011-026-05140-3

**Published:** 2026-05-16

**Authors:** Julia Araujo Alves, Priscila M. O. Muniz Cunha, Guilherme de Souza Meireles, Fábio Veríssimo Correia, André Luís de Sá Salomão, Rachel Ann Hauser-Davis, Enrico Mendes Saggioro

**Affiliations:** 1 Environmental Health Assessment and Promotion Laboratory (LAPSA), Oswaldo Cruz Institute (IOC), Oswaldo Cruz Foundation (Fiocruz), 4365 Brasil Avenue, Manguinhos, Rio de Janeiro, RJ Brazil; 2 Bioremediation and Phytotechnology Laboratory (LABIFI), Department of Environmental Engineering, Rio de Janeiro State University (UERJ), Rio de Janeiro, Brazil; 3 Environmental Health Laboratory, Department of Natural Sciences, Federal University of the State of Rio de Janeiro (UNIRIO), Rio de Janeiro, Brazil

**Keywords:** Environmental monitoring, *Oreochromis niloticus*, metal contamination, Subcellular distribution, urbanization

## Abstract

Urban coastal lagoons are heavily impacted by untreated sewage, industrial effluents and diffuse pollution, heightening ecotoxicological risks. Herein, Nile tilapia (*Oreochromis niloticus*) was employed as a sentinel species concerning metal exposure at the Jacarepaguá Lagoon Complex (JLC), a critical case of ecological degradation under intense anthropogenic pressure in Rio de Janeiro, Southeastern Brazil. Samplings were conducted between 2022 and 2023 during the dry (autumn–winter) and rainy (spring–summer) seasons. Organosomatic indices and total (muscle) and subcellular (liver and muscle) metal levels were determined. Antioxidant system alterations were evaluated through superoxide dismutase (SOD), glutathione-S-transferase (GST), reduced glutathione (GSH), total antioxidant capacity (TAC) and metallothionein (MT) assessments. Lipid peroxidation (LPO) and protein carbonylation (PTC) levels were also determined as oxidative effect markers, while vitellogenin (VTG) was used as an estrogenic effect indicator. Arsenic (0.018–0.033 mg L⁻¹), Mn (0.041–0.050 mg L⁻¹), Rb (0.019–0.023 mg L⁻¹), and V (0.008–0.010 mg L⁻¹) were detected in water samples in both sampling seasons; while Fe was detected only in the dry season (0.023 ± 0.03 mg L⁻¹). Subcellular As, Co, Cu, Fe, Mn, Ni, Pb, Rb, Ti, V, and Zn were quantified in both liver and muscle, albeit with no significant differences between seasons or sexes. Seasonal variations were noted for male VTG, although not statistically significant, with attenuation of sexual VTG differences from the dry (M/F = 0.67) to the rainy season (M/F = 1.20). The multibiomarker approach applied to wild tilapia under diffuse urban pollution indicates mild sublethal effects while highlighting the need for expanded, integrated monitoring of additional endocrine and oxidative biomarkers. Although centered on a specific lagoon complex system, these mechanisms recur in urban lagoons worldwide, indicating the need for consumption-risk evaluations and actionable remediation/management frameworks.

**Supplementary Information:**

The online version contains supplementary material available at 10.1007/s12011-026-05140-3.

## Introduction

Coastal environments are particularly vulnerable to the adverse effects of urbanization, resulting in water quality deterioration and consequent threats to biodiversity and ecosystem services [1, 2]. This situation is particularly critical in developing countries where suburban settlements, often driven by poverty and marked by critical infrastructure deficits, including inadequate sanitation systems and restricted access to potable water, pose a major challenge to environmental management [3, 4]. The typical lack of adequate urban infrastructure in these regions, such as stormwater drainage systems, intensifies the runoff and dispersal of contaminants into aquatic ecosystems [2, 5, 6].

In this context, the global expansion of urban areas without planning has led to substantial aquatic ecosystem impacts, primarily through the replacement of permeable surfaces with impervious ones, thereby increasing surface runoff and the influx of pollutants like metals, nutrients, and sediments into water bodies [3, 7]. This poses both ecological and human health risks, as many communities worldwide depend on coastal lagoons systems for food, recreation, and other ecosystem services [8].

Metals and metalloids comprise one of the main classes of chemical contaminants worldwide. Exposure to these compounds may disrupt the cellular redox balance, leading to the excessive generation of reactive oxygen species (ROS), which in turn can damage essential biomolecules, including proteins, lipids, and DNA, and impair metabolic functions [1, 9–11]. These responses are commonly assessed through oxidative stress biomarkers, such as antioxidant enzyme activities and oxidative damage indicators like lipid peroxidation (LPO), comprising sensitive tools for detecting early biological responses to pollutant exposure [12, 13]. Certain biomarkers of effect such as vitellogenin (VTG) induction, an endocrine disruption indicator, have also been applied in environmental monitoring assessments, especially in males [14]. Furthermore, integrative health metrics, including the condition factor (K) and organosomatic indices (e.g., hepatosomatic and gonadosomatic indices), offer insights into energy allocation, growth efficiency, and reproductive investment, aiding in elucidating sublethal impacts of complex pollutant mixtures on fish health and ecological fitness [15–17]. Several of these biomarkers have been employed in different taxa to assess the physiological impacts of contamination in environments affected by urban runoff, industrial effluents, and sediment-associated pollutants [9, 15, 18]. However, although metal bioaccumulation and oxidative stress biomarkers are commonly assessed in ecotoxicological assessments [19–22], most studies rely on simplified experimental conditions and fail to represent the chronic, multifactorial stress scenarios typical of urban aquatic ecosystems [22–25].

In this sense, Nile tilapia (*Oreochromis niloticus*) exhibits high commercial relevance, broad environmental tolerance and is widely distributed across tropical and subtropical regions Its bentho-pelagic behavior and dietary flexibility, however, lead to high susceptibility to xenobiotic accumulation from multiple exposure routes, making it a suitable sentinel species in urban aquatic systems [9, 26]. This species inhabits several Brazilian coastal lagoons, including the Jacarepaguá Lagoon Complex (JLC), a brackish system of high ecological relevance located in a densely urbanized area of Rio de Janeiro. Previous studies have reported intense anthropogenic pressure, spatial heterogeneity in water quality, and persistent nutrient enrichment in the system, highlighting the need for integrated assessments of contamination and biological effects. Alves et al., [15], Mendonça Ochs, [27], Souza, [28]. The JLC provides important ecosystem services, supporting artisanal and subsistence fisheries, flood and nutrient load regulation, carbon storage in mangroves, and cultural value through recreation and landscape appreciation [29]. Lagoon margins lie within the Atlantic Forest biome, surrounded by fragmented mangroves and a densely urbanized landscape ranging from informal settlements to high-income neighborhoods (Farias, [30]). Mean depths are generally shallow (< 3 m) across the complex, consistent with a system strongly controlled by morphology and restricted circulation ([31]; Santos, [32]). These hydrodynamic constraints, combined with high nutrient and pollutant loads due to untreated sewage discharge, industrial effluents, and diffuse urban pollution, contribute to hypereutrophic conditions, oxygen depletion events, and marked spatial gradients in salinity and water quality [31, 33, 34]. This, in turn, leads to recurrent cyanobacterial blooms, fish mortality events, invasive macrophyte proliferation, and persistent chemical contamination [27, 35, 36].

Despite its ecological importance, particularly its role in nutrient cycling, trophic regulation, and provision of ecosystem services such as fisheries and food supply for local communities [28], the magnitude of chemical contamination and its effects on aquatic fauna remain poorly understood. In this context, this study aimed to establish a reliable multibiomarker approach to generate ecotoxicological evidence under real environmental conditions, contributing to a comprehensive assessment of chronic stress in Nile tilapia inhabiting the JLC. This approach is applicable to other coastal areas in Brazil, and extensible worldwide.

## Materials and Methods

### Study Area

**Fig. 1:**
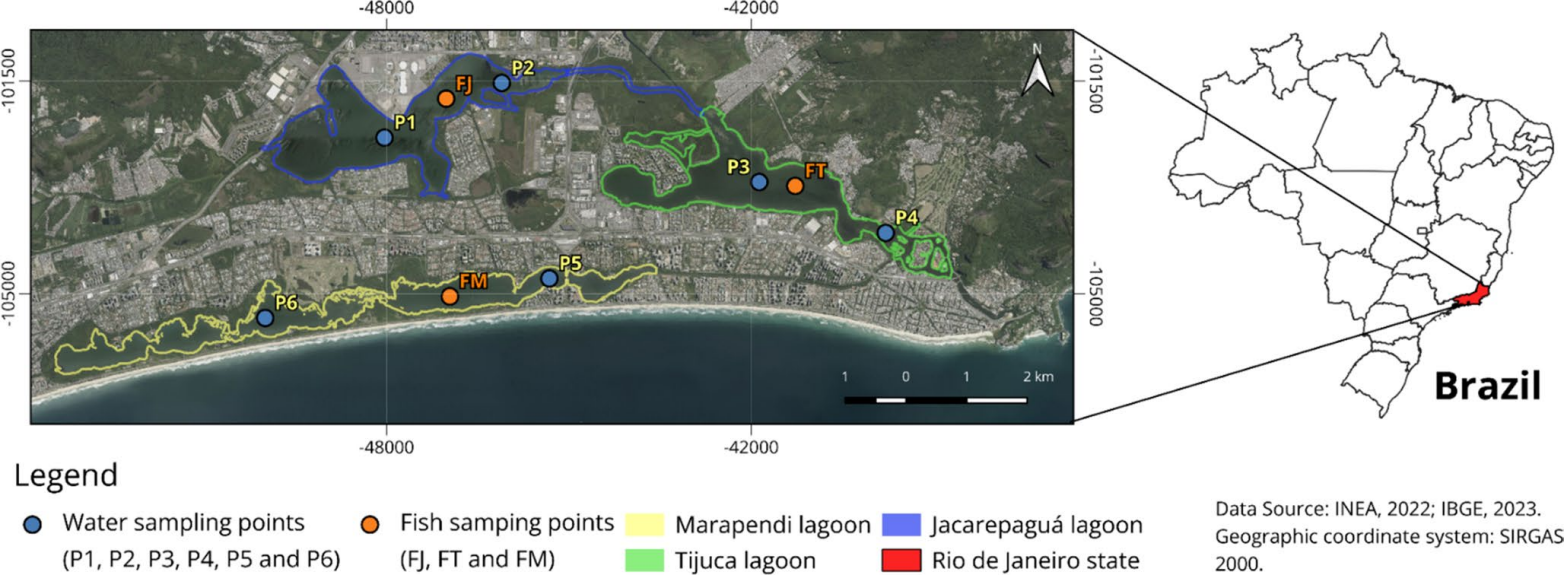
Geographic location of the Jacarepaguá Lagoon Complex (JLC), southeastern Brazil. Water-sampling points are shown in blue (P1–P6) and fish-sampling points in orange (FJ, FT, FM). The map delineates the Jacarepaguá (blue line), Tijuca (green line), and Marapendi (yellow line) lagoons within the city of Rio de Janeiro. Insets show the JLC within Rio de Janeiro state and Brazil

The Jacarepaguá Lagoon Complex (JLC), located in the western zone of Rio de Janeiro, southeastern Brazil, comprises interconnected coastal lagoons: Jacarepaguá, Tijuca, Camorim and Marapendi (Fig. 1). The complex covers approximately 13.3 km² and drains a watershed of about 280 km² [37]. Jacarepaguá Lagoon, the most inland unit (~ 4.07 km²), receives substantial freshwater inputs from urban tributaries, particularly the Pavuna and Arroio Pavuna rivers [34]. Tijuca Lagoon (~ 4.34 km²) functions as an intermediate compartment with restricted hydrodynamic exchange [38]. Camorim Lagoon, the smallest unit (~ 0.80 km²), acts primarily as a hydrodynamic connector between Jacarepaguá and Tijuca lagoons [34]. Marapendi Lagoon, the most seaward basin, is elongated parallel to the coastline, presents the largest surface area (~ 4.3 km²), and exhibits the widest connection to marine waters via the Joatinga Channel [39].

### Sampling Design

Samplings were conducted between 2022 and 2023, covering both the dry (autumn and winter) and rainy (spring and summer) seasons, typical of southeastern Brazil [40]. To capture the spatial variability of the lagoon complex, surface water samples were collected from six georeferenced sites (P1–P6) during four campaigns (January, April, July, and October). The sampling stations were selected to represent distinct environmental conditions and anthropogenic influences across the system.

Site P1 (664335.20 mE; 7457061.26 mN) is located central region of Jacarepaguá Lagoon, receives untreated domestic effluents and is influenced by both the Arroio Pavuna River and the Cortado Canal. P2 (667431.03 mE; 7458163.35 mN) is characterized by low hydrodynamic flow and sediment retention. P3 (670495.24 mE; 7458661.10 mN) is located near densely populated areas, including the Stones River (~ 23,846 households) and Muzema (~ 12,982 inhabitants) [41]. P4 (671942.65 mE; 7455643.86 mN), near Gigóia island, is influenced by wastewater inputs from the surrounding urban areas with limited sewage infrastructure. Sites P5 (667407.61 mE; 7458482.12 mN) and P6 (659069.33 mE; 7453622.46 mN) are located closer to the coastal zone and are influenced by marine inputs, resulting in higher salinity.

A total of 10 L of surface water (5–10 cm in depth) was obtained from each site using specific containers for each type of analysis, following [42] and national protocols ANA [43]. Nile tilapia specimens were sampled at fixed sites within Jacarepaguá, Tijuca, and Marapendi lagoons using 10 casts of a circular net (12.0 m perimeter and 30.0 mm mesh). Given the mobility and bentho-pelagic behavior of O. niloticus, which allows individuals to integrate exposure across different areas of the lagoon system, and considering the study’s focus on an integrated, system-level assessment, data were analyzed in a pooled manner to reflect overall environmental conditions.

### Water Analysis

Water quality was assessed by determining physicochemical and microbiological parameters in situ using a multiparameter probe (Horiba U-50) for pH, redox potential (ORP), dissolved oxygen (DO), temperature (Temp.), turbidity (Turb.), salinity (Sal.), electrical conductivity (EC), and total dissolved solids (TDS). Other physicochemical analyses followed [42] protocols, namely Chemical Oxygen Demand (COD) by method 5220 D; total phosphorus (P) using Hach TNT 843/844 kits (USEPA-approved) and total nitrogen (N) via the 4500-N method. The BOD/COD ratio was fixed at 0.5 for all stations, based on literature values [44]. Thermotolerant coliforms (TtC) and *Escherichia coli* were quantified using the Colilert^®^ Idexx colorimetric analysis kit, with results expressed as MPN 100 mL^− 1^. All samples were stored in a thermal box containing ice until laboratory arrival [42].

The Water Quality Index (WQI), adapted from the Brazilian National Sanitation Foundation (NSF) and Brazilian National Water Agency [45], was applied, integrating nine parameters with assigned weights: DO (0.17), EC (0.15), pH (0.12), BOD (0.10), temperature (0.10), total nitrogen (0.10), total phosphorus (0.10), turbidity (0.08), and TDS (0.08). The WQI values were interpreted according to five qualitative categories: ≤19 (very poor), 20–36 (poor), 37–51 (fair), 52–79 (good), and 80–100 (excellent). Following Eq. 1, where *q* represents the quality score of each parameter (0–100), *w* the weight assigned to each, and *n* the number of variables included:

1 $$WQI = \sum_n^n \cdot q_{n} \times W_{n}$$

### Fish Analyses

Sixty-eight Nile tilapia were collected from a representative site at each lagoon (32 dry and 36 rainy seasons). Mean weight and total length were higher in the rainy season (males: 288.76 ± 41.06 g and 25.69 ± 1.37 cm; females: 301.34 ± 57.95 g and 25.29 ± 2.29 cm) than in the dry season (males: 197.39 ± 81.52 g and 21.21 ± 4.22 cm; females: 215.05 ± 77.69 g and 22.63 ± 4.03 cm). Blood was sampled via the caudal vein using BD Ultra-Fine™ syringes (50 U, 12.7 mm × 0.33 mm; New Jersey, USA), kept in heparinized microtubes (150 IU mL⁻¹) and chilled during transport. At the laboratory, plasma was separated by centrifugation at 1,200 × g for 10 min and subsequently used for VTG quantification through alkaline phosphatase (ALP) activity assays.

Euthanasia was performed by ice slurry immersion (hypothermia), followed by decapitation [46]. Total lengths and body weights were recorded for each individual, followed by sex determination and internal organ dissection. Liver and gonads were excised, weighed to calculate organosomatic indices and stored at − 80 °C for oxidative stress biomarker and intracellular metal quantifications. All fish handling procedures were approved by the Chico Mendes Institute for Biodiversity Conservation (SISBIO license n° 87698-1>), the National System for the Management of Genetic Heritage and Associated Traditional Knowledge (SISGEN license nº A5C3171), and the Rio de Janeiro State University Ethics Committee on the Use of Animals (protocol nº. 021/2018).

The Hepatosomatic Index (HSI) and Gonadosomatic Index (GSI) were calculated using Eqs. 2 and 3, respectively, where *Wh* and *Wg* represent liver and gonad weight (g), and *Wt* is the total body weight (g). The GSI was calculated only for mature or maturing individuals [17]. The Fulton Condition Factor (K) was also estimated as a proxy for nutritional status [47], using total weight (Wt) and total length (Lt), as depicyed in Eq. 4.

2 $$\:HSI=\frac{Wh}{Wt}*100$$

 

3 $$\:GSI=\frac{Wg}{Wt}*100$$

 

4 $$\:K=\frac{Wt}{{Lt}^{3}}*100$$

 

### Biomarker Analyses

Liver and muscle aliquots (50 mg) were homogenized (1:4, m/v) in 0.1 mol L⁻¹ sodium phosphate buffer (pH 6.5) containing 0.25 mol L⁻¹ sucrose and 1 mmol L⁻¹ EDTA, and centrifuged at 20,000 × g for 30 min at 4 °C (Eppendorf 5430R). The supernatants were used for antioxidants system and oxidative damage analysis. Sample dilutions varied by assay: liver extracts were diluted 50× for SOD, MT and GSH; 10× for TAC; 5× for LPO and PTC. Muscle extracts were diluted 40× for SOD, MT and GSH; 5× for TAC; 2× for LPO and PTC, while GST was measured in undiluted samples for both tissues. Protein concentrations were determined according to [48], modified by [49], and used to normalize biomarker data.

Superoxide dismutase (SOD) activity was measured using a commercial kit (Cayman Chemical Company, USA), which determines the three SOD types (Cu/Zn-SOD, Mn-SOD and Fe-SOD), based on superoxide radical (O_2_^−^) detection. The method uses tetrazolium salt (200 µL) to detect radicals generated in tissues samples (10 µL) in the presence of xanthine oxidase (20 µL), with 30 min of dark incubation. Absorbances were determined at 450 nm on INNO microplate reader (LTEK Co., Moita, Portugal). Results were expressed as U mg⁻¹ ptn (international enzyme activity units normalized to total protein).

Glutathione S-transferase (GST) activity followed the method of Habig et al., [50], based on the conjugation of reduced glutathione (GSH; 30 mmol.L^− 1^) in the presence of 1-chloro-2,4-dinitrobenzene (CDNB; 30 mmol.L^− 1^), resulting in S-(2,4-Dinitrophenyl)-glutathione (GS-DNB). Absorbances were determined for 60 s at 340 nm using a Bel UV-M51 Vis spectrophotometer (Bel Engineering, Italy), and activity was expressed as U mg⁻¹ ptn.

Reduced glutathione (GSH) content was quantified with 2,2′-dinitro-5, 5′-dithiodibenzoic acid (DTNB) as described by Wilhelm Filho et al., [51]. Samples were incubated with DTNB (0.25 mmol L⁻¹), at a 1:1 ratio for 15 min and absorbances were determined at 412 nm using INNO microplate reader (LTEK Co., Moita, Portugal). Results were expressed as µmol mg⁻¹ ptn.

Total antioxidant capacity (TAC) was determined using 2,2’-azinobis (3-ethylbenzothiazoline-6-sulfonic acid) diammonium salt (ABTS, 7 mmol L⁻¹) radical method and potassium persulfate (K₂S₂O₈, 2,45 mmol L⁻¹) [52], with Trolox (2 mmol L⁻¹) as the standard. Absorbances were determined at 734 nm INNO microplate reader (LTEK Co., Moita, Portugal) and results reported as mmol mg⁻¹ ptn.

Metallothionein (MT) quantification followed Erk et al., [53]. Tissues (150 mg) were homogenized in Tris buffer (19 mmol L⁻¹, pH 8.6), with phenylmethanesulfonyl fluoride (PMSF, 28 mmol L⁻¹) and β-mercaptoethanol (14 mmol L⁻¹). Supernatants were separated by centrifugation at 20,000 × g for 60 min at 4 °C on an Eppendorf 5430R centrifuge (Hamburg, Germany), thermally treated at 70 °C for 10 min, and centrifuged again. Quantification used Ellman’s reagent (EDTA 4 mmol L^− 1^ and DTNB 0.43 mmol.L^− 1^ in 2 mol.L^− 1^ sodium chloride) and followed the Ellman method [54], with absorbance at 412 nm after incubation for 15 min in the dark on INNO microplate reader (LTEK Co., Moita, Portugal). Results were expressed as µmol mg⁻¹ ptn, adopting the 1:20 for MT: GSH molar ratio, as described by Kägi [55]. Aliquots of the final purified supernatant (100 uL) were used for intracellular muscle and liver metal determinations.

Lipid peroxidation (LPO) was determined as malondialdehyde (MDA) equivalents via the thiobarbituric acid (TBA) reaction [56]. Tissues (100 mg) were homogenized in extraction buffer containing 0.5 mL of Triton X-100, 2.5 mL of 1 mol L⁻¹ Tris-HCl (pH 7.5), 2.5 mL of 3 mol L⁻¹ NaCl, 0.05 g of sodium dodecyl sulfate (SDS) and 0.5 g of sodium deoxycholate (1:4, m/v), centrifuged at 1,600 × g for 10 min at 4 °C and incubated at 100 °C for 60 min. Absorbances were measured at 535 nm on INNO microplate reader (LTEK Co., Moita, Portugal) and results expressed as µmol MDA mg⁻¹ ptn.

Protein carbonylation (PTC) levels were assessed using the 2,4-dinitrophenylhydrazine (DNPH) method [57]. Liver and muscle supernatant were incubated with 10 mmol L⁻¹ DNPH prepared in 0.5 mol L⁻¹ phosphoric acid (H₃PO₄) for 10 min. Absorbances were determined at 450 nm on an INNO microplate reader (LTEK Co., Moita, Portugal) and results were expressed as µmol mg⁻¹ ptn.

Vitellogenin (VTG) was used as an endocrine disruption biomarker via the alkali-labile phosphate (ALP) method, as described by Gagné and Blaise [58] with modifications by Hallgren et al., [59]. Plasma samples (100 µL) were precipitated with 54 µL of 35% acetone (v/v) and centrifuged at 5,000 × g for 5 min. Pellets were incubated with 100 µL of 1 mol L^− 1^ NaOH at 70 °C for 90 min and re-precipitated with 40 µL of 100% TCA. After centrifugation at 20,000 × g for 5 min, supernatant (105 µL) was mixed with 435 µL of distilled water and equal volume of ≥ 99% 1-butanol. PO₄³⁻ was quantified by 70 µL of 1% ammonium molybdate in 2 mol L^− 1^ H₂SO₄ and equal volume of 5% ascorbic acid incubated for 60 min at 40 °C. Absorbance was measured at 630 nm on INNO microplate reader (LTEK Co., Moita, Portugal) and results were expressed as µg PO₄³⁻ mL⁻¹ of plasma.

### Metal and Metalloid Analyses

Metal and metalloid concentrations in water (*n* = 24) and fish (*n* = 68) samples were determined by Inductively Coupled Plasma Mass Spectrometry (ICP-MS) using a NexION 300X instrument (PerkinElmer, USA). Water samples (100 mL) were collected in pre-acidified sterile high-density polypropylene (HDPE) tubes (1.0 mL of HNO_3_ 67% v/v; Vetec, Brazil) and analyzed without additional treatment. For fish samples, 0.100 g of muscle (total fraction) or metallothionein (MT) extract (intracellular fraction) were transferred to 15 mL polypropylene tubes, digested overnight with 1.0 mL sub-boiled HNO_3_, followed by heating at 100 °C for 6 h. Digests were diluted to 10 mL with ultrapure water.

External multielement calibration curves were used and rhodium (¹⁰²Rh) was an internal standard. Accuracy was evaluated through procedural blanks and a certified reference material (DORM-5; NIST 2976; BCR 668 Mussel Tissue; European Commission), processed in triplicate alongside the samples. All calibration curves showing correlation coefficients > 0.995. Recoveries ranged from 60 to 135%, which we consider acceptable for these matrices and the intended assessment (Table S1, Supplementary Material). Results were expressed in mg L⁻¹ (water) and mg kg^− 1^ wet weight (w.w.) (fish). Instrumental Limits of Detection (LOD) and method Quantification (LOQ) were calculated according to [60] are listed in Table S2 (Supplementary Material).

### Human Health Risk Assessments

Human health risk assessment was performed to estimate potential non-carcinogenic and carcinogenic risks associated with tilapia muscle consumption. The following indices were calculated: Estimated Daily Intake (EDI), Estimated Weekly Intake (EWI), Target Hazard Quotient (THQ), Hazard Index (HI), and Target Cancer Risk (TCR). Analyses were performed separately for females and males across the following age groups: (i) infants (7–12 months; fish introduction recommended at ≥ 6 months according to [61], (ii) children (3–11 years), and (iii) adults (19–75 years); body weights (BWa) for each age group [62]. Consumption scenarios simulated fish intake from one to five meals per week. For all elements, the Estimated Daily Intake (EDI) was calculated as Eq. 5:

5 $$\:EDI=\frac{C\times\:FIR}{BWa}$$

 

 Where C is the concentration in tilapia muscle (mg kg⁻¹, wet weight), FIR is the daily fish ingestion rate per person (kg day⁻¹), and BWa is the reference body weight (kg). Weekly intake (EWI) was calculated by multiplying the EDI by 7.

The Target Hazard Quotient (THQ) was calculated following US EPA guidance [63]. It represents the ratio between the estimated exposure dose to a given element and its oral reference dose (RfD), the level below which non-carcinogenic effects are not expected [63]. THQ < 1 indicates no expected concern; THQ > 1 suggests potential adverse effects [64], calculated as Eq. 6.

6 $$\:THQ=\left(\frac{EFR\:\times\:Ed\times\:FIR\times\:C}{RfD\:\times\:BWa\:\times\:ATn}\right)\times\:{10}^{-3}$$

 

 Where EFR is the exposure frequency to a certain metal or metalloid, ED is exposure duration for each age category, FIR is the foodstuff intake rate (g day^− 1^), C is the wet weight concentration of a certain element in the given food item, RfD is the reference oral dose of the determined element (µg g^− 1^ day^− 1^), according to the US EPA [63], BWa is the reference body weight of each evaluated age group (infants, children and adults), ATn is the average exposure time (365 days × Ed) and 10^− 3^ is a conversion factor unit. The RfDs used for each element were as follows: As 3 × 10^− 4^ µg ⁻¹ day⁻¹, Cd 1 × 10^− 3^ µg ⁻¹ day⁻¹, Cu 4 × 10^− 3^ µg ⁻¹ day⁻¹, Ni 112 10^− 4^ µg ⁻¹ day⁻¹, Se 5 × 10^− 3^ µg ⁻¹ day⁻¹, V 9 × 10^− 3^ µg ⁻¹ day⁻¹ and Zn 0.3 µg ⁻¹ day⁻¹ [63].

The Hazard Index (HI) represents the aggregate non-cancer risk from multiple elements, computed as the sum of the individual THQs for each metal/metalloid detected. It assumes that consuming the food leads to concurrent exposure to several potentially toxic elements. HI > 1 signals a potential for adverse health effects, whereas values ≤ 1 suggest no expected concern. This index is calculated as Eq. 7:

7 $$\:HI={\sum\:}_{n=1}^{i}{THQ}_{n}$$

 

The target cancer risk (TCR) determines the probability of excess cancer risk over the lifetime of exposed individuals for elements with established oral cancer slope factors (CPSO) [65–67]. TCR was computed as Eq. 8:

8 $$\:TCR=\left(\frac{EFR\:\times\:Ed\times\:FIR\times\:C\times\:CPSO}{BWa\:\times\:ATc}\right)\times\:{10}^{-3}$$

 

 where AT₍c₎ is the averaging time for carcinogenic effects; and 10⁻³ is a unit conversion factor. We adopted CPSO values of 1.5 mg·kg⁻¹·day⁻¹ for iAs, 1 × 10⁻³ mg·kg⁻¹·day⁻¹ for Cd, 8.5 × 10⁻³ mg·kg⁻¹·day⁻¹ for Pb and 5 × 10⁻³ mg·kg⁻¹·day⁻¹ for Se [65–67]. For arsenic, two inorganic-fraction scenarios were applied: best case (1% iAs) and worst case (10% iAs)—reflecting typical ranges of the toxic inorganic fraction in fish muscle. TCR values > 1 × 10⁻⁴ were interpreted as indicating unacceptable carcinogenic risk.

### Statistical Analyses

Biomarker outliers were screened using the ROUT method (Q = 1%). Normality was assessed using the Shapiro-Wilk test. For each endpoint, the four groups (Male–Dry, Female–Dry, Male–Rainy, Female–Rainy) were compared with a Kruskal–Wallis test for biochemical parameters, metal concentrations and biomarker responses with Dunn’s *post hoc* test. As rank-based methods were used, homogeneity of variances was not formally assessed. Fish results are reported as medians and ranges (min-max), whereas water data are reported as mean ± SD. Results were considered statistically significant at *p* < 0.05. When sex did not influence the seasonal pattern, data were pooled across sex. Separate results were shown only when sex effects were evident. All analyses were conducted in GraphPad Prism^®^ 8.0.

A Principal Component Analysis (PCA) was applied to explore biomarker patterns in fish tissues using the vegan and ggplot2 packages in the R environment (version 2025.05.0 + 496). Prior to PCA, data suitability was assessed using Bartlett’s test of sphericity and the Kaiser–Meyer–Olkin (KMO) measure of sampling adequacy. It should be noted that Bartlett’s test was used exclusively to evaluate the adequacy of the correlation matrix for PCA, and not to assess homogeneity of variances between groups. Only the liver dataset met the assumptions for PCA, presenting a significant Bartlett’s test (*p* < 0.05) and an overall KMO value of 0.53, indicating moderate but acceptable sampling adequacy. The muscle dataset did not meet these criteria and was therefore excluded from multivariate analysis. PCA was performed exclusively on liver biomarkers using the prcomp function, with centered and scaled variables. Interpretation of the PCA was based on the proportion of variance explained by the principal components, variable loadings, and squared cosines (cos²).

## Results and Discussion

### Water Parameters

Physicochemical and microbiological parameters showed considerable variability across the monitoring campaigns (Table 1). The study period encompassed both the dry (autumn and winter) and rainy (spring and summer) seasons, with mean precipitation of approximately 712.4 mm during the rainy season and 79.2 mm during the dry season, consistent with the regional climatic pattern described for southeastern Brazil [40].

**Table 1:** Physicochemical, microbiological parameters and Water Quality Index (WQI) of water samples from seasonal campaigns (2022–2023) at the Jacarepaguá Lagoon Complex (JLC), Rio de Janeiro, Southeastern Brazil. *asteriscks indicate single values. NA- not available values

Sampling sites		pH	DO (mg/L)	EC (mS/cm²)	Turb. (NTU)	Temp. (ºC)	ORP (mV)	TDS (mg/L)	Sal. (ppt)	COD (mg/L)
Rainy Season	**P1**	8.55 ± 0.78	8.65 ± 4.31	2.20 ± 1.98	103.70 ± 48.51	26.05 ± 1.91	45.75 ± 55.51	1011.50 ± 1414.92	1.15 ± 1.06	171.15 ± 63.99
	**P2**	8.10 ± 0.99	6.25 ± 3.32	3.20 ± 2.97	57.35 ± 13.79	26.25 ± 2.05	41.80 ± 47.52	1589.00 ± 1600.89	1.65 ± 1.63	164.90 ± 27.72
	**P3**	7.35 ± 0.21	4.45 ± 1.48	7.15 ± 8.70	50.40 ± 8.77	25.55 ± 1.91	37.25 ± 38.25	3690.00 ± 4577.81	4.05 ± 5.02	234.50 ± 72.69
	**P4**	7.85 ± 0.21	8.80 ± 0.71	11.80 ± 13.01	27.00 ± 17.82	26.55 ± 1.91	46.05 ± 55.08	7382.50 ± 8467.60	8.95 ± 10.54	675.40 ± 520.15
	**P5**	7.70 ± 0.28	7.55 ± 2.33	22.90 ± 13.72	6.80 ± 4.95	24.30 ± 1.70	5.30*	11508.50 ± 6578.21	14.05 ± 8.70	961.15 ± 647.78
	**P6**	7.55 ± 0.49	6.50 ± 5.52	6.60 ± 4.53	39.60 ± 7.21	23.75 ± 1.77	5.80*	3301.50 ± 2260.62	3.60 ± 2.55	306.90 ± 30.55
Dry Season	**P1**	8.05 ± 0.64	12.90 ± 8.77	41.20 ± 49.78	131.50 ± 6.36	21.05 ± 3.18	56.00 ± 63.64	4305.00 ± 714.18	2.20 ± 2.83	562.35 ± 79.69
	**P2**	7.90 ± 0.14	8.85 ± 3.61	41.00 ± 49.64	119.15 ± 28.07	22.00 ± 2.97	33.50 ± 3.54	4240.00 ± 791.96	4.20*	479.00 ± 13.72
	**P3**	7.40 ± 0.14	6.40 ± 6.36	44.65 ± 52.54	105.30 ± 12.30	21.40 ± 2.55	NA	4830.00 ± 466.69	2.70 ± 2.69	612.35 ± 27.79
	**P4**	8.50 ± 0.00	14.45 ± 0.64	84.00 ± 97.58	111.00 ± 12.73	22.80 ± 2.97	NA	9475.00 ± 360.62	6.40 ± 3.11	854.00 ± 125.44
	**P5**	7.00 ± 0.57	7.65 ± 6.29	314.00*	163.10 ± 200.68	25.30 ± 3.68	NA	19200.00*	20.60*	1292.35 ± 28.78
	**P6**	7.00 ± 0.57	6.00 ± 8.49	116.00*	220.50 ± 102.53	25.95 ± 3.18	39.00*	7180.00*	10.10*	1165.65 ± 376.68
		BOD * (mg/L)	*P*(mg/L)	PO₄³⁻ (mg/L)	*N*(mg/L)	TtC(NMP/100mL)	E. coli (NMP/100mL)	WQI	Class.	
Rainy Season	**P1**	34.25 ± 12.80	2.00 ± 0.28	NA	32.70 ± 4.93	25352.44 ± 23953.15	1707.17 ± 1198.07	27.70 ± 1.84	Poor	
	**P2**	33.00 ± 5.52	1.80 ± 0.85	NA	28.60 ± 26.59	177225.70 ± 185715.51	59197.71	25.75 ± 3.46	Very poor	
	**P3**	46.90 ± 14.57	1.80*	NA	13.30 ± 5.37	193104.55 ± 257116.12	36168.60 ± 50819.20	29.75 ± 9.97	Poor	
	**P4**	135.05 ± 104.02	1.00 ± 0.57	NA	10.20 ± 5.23	79551.25 ± 110273.66	15233.20 *	36.95 ± 13.79	Poor	
	**P5**	192.20 ± 129.54	1.45 ± 1.20	NA	15.00 ± 5.37	70172.05 ± 85617.97	31626.10 ± 44515.34	34.80 ± 11.31	Poor	
	**P6**	61.40 ± 6.08	2.85 ± 0.49	NA	22.95 ± 11.67	6312.30 ± 5505.11	201616.55 ± 284393.47	26.50 ± 9.33	Poor	
Dry Season	**P1**	112.45 ± 15.91	77.75 ± 103.87	25.31 ± 33.79	131.60*	NA	2065.00 ± 275.77	0.10 ± 0.14	Very poor	
	**P2**	95.80 ± 2.69	83.95 ± 111.51	27.43 ± 36.44	24.20*	NA	44545.00 ± 59531.32	0.10 ± 0.14	Very poor	
	**P3**	122.45 ± 5.59	41.25 ± 49.71	13.40 ± 16.13	13.00*	101.12*	2595.98 ± 3329.09	0	Very poor	
	**P4**	170.80 ± 25.03	96.00*	31.30*	16.40*	NA	7320.00 ± 4794.18	22.70 ± 9.76	Very poor	
	**P5**	258.45 ± 5.73	24.90*	27.30*	128.00*	3637.31 ± 4995.98	360.00 ± 226.27	29.00 ± 15.84	Poor	
	**P6**	233.15 ± 75.31	46.00*	14.90*	24.60*	NA	3410.00 ± 2262.74	25.70 ± 17.57	Very poor	

*DO* Dissolved Oxygen, *EC* Electrical Conductivity, *Turb* turbidity, *Temp* temperature, *ORP* Oxi-reduction Potential, *TDS* Total Dissolved Solids, *Sal* salinity, *COD* Chemical Oxygen Demand, *BOD* Biological Oxygen Demand, *P* Phosphorus, *PO*₄³⁻ Phosphate, *N* Total Nitrogen, *TtC* Thermotolerant Coliform, *E. coli* Escherichia coli, *WQI* Water Quality Index, *Class* Classification. * BOD estimated from the BOD/COD ratio. with a value of 0.5 for the sampling points (Bollmann e Marques. 2006). The values in bold are above the limit established by resolution CONAMA nº 357 (2005) which provides for the classification of water bodies and environmental guidelines for their classification. as well as establishing the conditions and standards for the release of Brazilians effluents

According to Brazilian legislation [68], the JLC is classified as brackish water Class 3, designated for activities such as navigation and secondary contact recreation. However, several parameters exceeded the established limits for this class, as follows: dissolved oxygen (DO) ranged from 0 to 19.1 mg L⁻¹ (mean 8.2 ± 4.63 mg L⁻¹), with values below the minimum recommended limit (> 3.0 mg L⁻¹) at multiple sites and time points. pH values ranged from 6.6 to 9.1 (mean 7.75 ± 0.62), remaining mostly within the acceptable range (5.0–9.0). In contrast, turbidity varied from 3.3 to 305 NTU (mean 94.62 ± 77.51 NTU), frequently exceeding the recommended limit of 100 NTU, particularly during the dry season. Biochemical oxygen demand (BOD) values were consistently above the acceptable limit (< 10 mg L⁻¹) in all samples, with mean values higher during the dry season (165.52 ± 68.86 mg L⁻¹) than during the rainy season (83.80 ± 80.81 mg L⁻¹). In a previous study conducted in the same area in October 2022, Corrêa-Moreira et al., [69] reported similar conditions, indicating a continuous high ecological risk. Although *E. coli* is not explicitly regulated under CONAMA 357/05 [68], thermotolerant coliforms counts frequently exceeded the guideline value of > 4000 MPN/100 mL (CONAMA 274/2000) [70], with means of 58976.20 ± 118746.61 MPN/100 mL (rainy season) and 30192.50 ± 70909.69 (dry season). These values are substantially higher than those reported for other Brazilian urban lagoons, such as Rodrigo de Freitas Lagoon, where coliform levels ranged from 930 to 24,000 MPN/100 mL [71]. These conditions also represent indirect risks to human health through deteriorating water quality and potential fish contamination. Eutrophication-related factors, particularly nutrient enrichment and reduced DO levels, create favorable conditions for microbial proliferation in aquatic environments [69].

Surface water quality indicators further reflect the critical environmental status of the JLC. The Water Quality Index (WQI), calculated according to the Brazilian National Water Agency [45], ranged from 0 to 46.74, corresponding to the “poor to very poor” categories. The decline during the dry season highlights the role of reduced hydrological renewal in altering water-quality parameters and favoring pollutant concentration. As the WQI was originally developed for freshwater systems, its application in brackish conditions should be interpreted with caution, particularly for salinity-influenced parameters (e.g., conductivity and TDS), and is therefore considered here as a comparative indicator. Altogether, the results reinforce JLC as a highly impacted urban aquatic system.

### Metal and Metalloid Levels in Water

Metal and metalloid concentrations in surface water from the JLC are presented in Table 2. Arsenic, Mn, Rb and V were detected in both the dry and rainy periods, with mean concentrations (rainy and dry season, respectively) as follows: As = 0.0183 ± 0.0117 and 0.0325 ± 0.0126; Mn = 0.0500 ± 0.0501 and 0.0410 ± 0.0348; Rb = 0.0229 ± 0.0205 and 0.0193 ± 0.0166; V = 0.00822 ± 0.00908 and 0.0103 ± 0.00742 mg L⁻¹. Iron and Zn were detected only in the dry season with values 0.0300 mg L⁻¹ and 0.345 ± 0.0354 mg L⁻¹ respectively while Hg was detected only in the rainy season (0.0100 mg L⁻¹).

**Table 2:** Concentrations of metals and metalloids (mg L⁻¹) in surface water samples collected from the Jacarepaguá Lagoon Complex (JLC) during dry and rainy season. Values below the limit of quantification are indicated as < LQ. *asteriscks indicate single values

	Dry Season	Rainy Season
Elements	Mean ± SD (min-max)	Mean ± SD (min-max)
As	0.0183 ± 0.0117 (0.01 − 0.04)	0.0325 ± 0.0126 (0.02 − 0.05)
Fe	0.345 ± 0.0354 (0.32 − 0.37)	< LQ
Hg	< LQ	0.0100 *
Mg	215 ± 209 (57.2–746)	116 ± 143 (7. 54–487)
Mn	0.0500 ± 0.0501 (0.01 − 0.15)	0.0410 ± 0.0348 (0.01 − 0.110)
Rb	0.0229 ± 0.0205 (0.005 − 0.073)	0.0193 ± 0.0166 (0.005 − 0.058)
V	0.00822 ± 0.00908 (0.001 − 0.03)	0.0103 ± 0.00742 (0.003 − 0.024)
Zn	0.0300 *	< LQ

These findings align with a scenario of chronic environmental disturbance in the system, marked by recurrent eutrophication ([72]; [73], relatively high metal loadings [15, 34, 74], sediment infilling [75, 76], continuous effluent and solid waste discharges due to rapid, poorly planned urbanization [77]. Reported anthropogenic inputs of Zn, Cu, Pb, and Hg (mean values = 176.9 ± 91.6, 45.1 ± 21.3, 35.2 ± 15.0, 0.1442 ± 0.0893 mg kg^− 1^) in Tijuca Lagoon, an area that corresponds to the influence zone of sampling sites P3 and P4 in the present study, supporting the occurrence of contamination in this region (linked to industrial residues, improperly disposed solid waste, urban runoff and domestic sewage).

The authors emphasize that, beyond metals and hydrocarbons, biological responses likely reflect eutrophication and the presence of unmonitored contaminants such as hormones, pharmaceuticals, Pharmaceuticals and Personal Care Products (PPCPs), Per- and Polyfluoroalkyl Substances (PFAS), and detergents [34].

Arsenic is often linked to industrial effluents, agricultural runoff (pesticides/fertilizers), and coal/oil combustion, being transported by stormwater and atmospheric deposition [78]. Vanadium in urban lagoons typically reflects mixed inputs. Major sources include fuel-oil combustion from vessels, appearing as fine particulate matter in navigation channels and port areas [79], as well as emissions from refineries, fuel terminals, thermoelectric plants, and metallurgical activities [80]. Urban drainage can further transport particulate material enriched with metals into JLC after rainfall. The higher V concentrations observed during the rainy campaign likely reflect enhanced mobilization and transport of V associated with urban runoff and atmospheric deposition, rather than increased primary emissions from navigation and industrial activities [79].

Essential metals such as Mn and Fe occur naturally and mainly reflect local geology, redox regime, and dissolved organic matter [81]. However, although these elements are biologically essential for processes such as enzymatic activity, photosynthesis, and electron transport, excess concentrations may become toxic [82]. In estuarine environments, Fe and Mn undergo redox cycling (Fe³⁺/Fe²⁺; Mn⁴⁺/Mn²⁺), promoting coprecipitation, colloidal aggregation, or complexation with organic matter [83, 84]. Rubidium (Rb), on the other hand, is predominantly of geogenic origin, commonly associated with the weathering of K-rich minerals [85]. However, its increasing use in electronic devices, specialty glasses, and advanced technologies has led to the recognition of Rb as a Technologically Critical Element [85]. Although natural sources still dominate its global geochemical cycle, electronic waste, industrial activities, and fossil fuel combustion have emerged as relevant secondary anthropogenic sources of Rb, particularly in urbanized and industrialized environments [86–89]. These sources may contribute to local alterations in Rb concentrations through atmospheric deposition, urban runoff, and sediment remobilization in aquatic systems.

In the case of shallow aquatic environments, it is also important to emphasize that sediments can be a constant secondary source of exposure, with diffusion and resuspension maintaining the supply of metals with or without new point transport [81, 90].

### Metal and Metalloid Levels in Fish

#### Total Metal Concentrations in Muscle

**Fig. 2:**
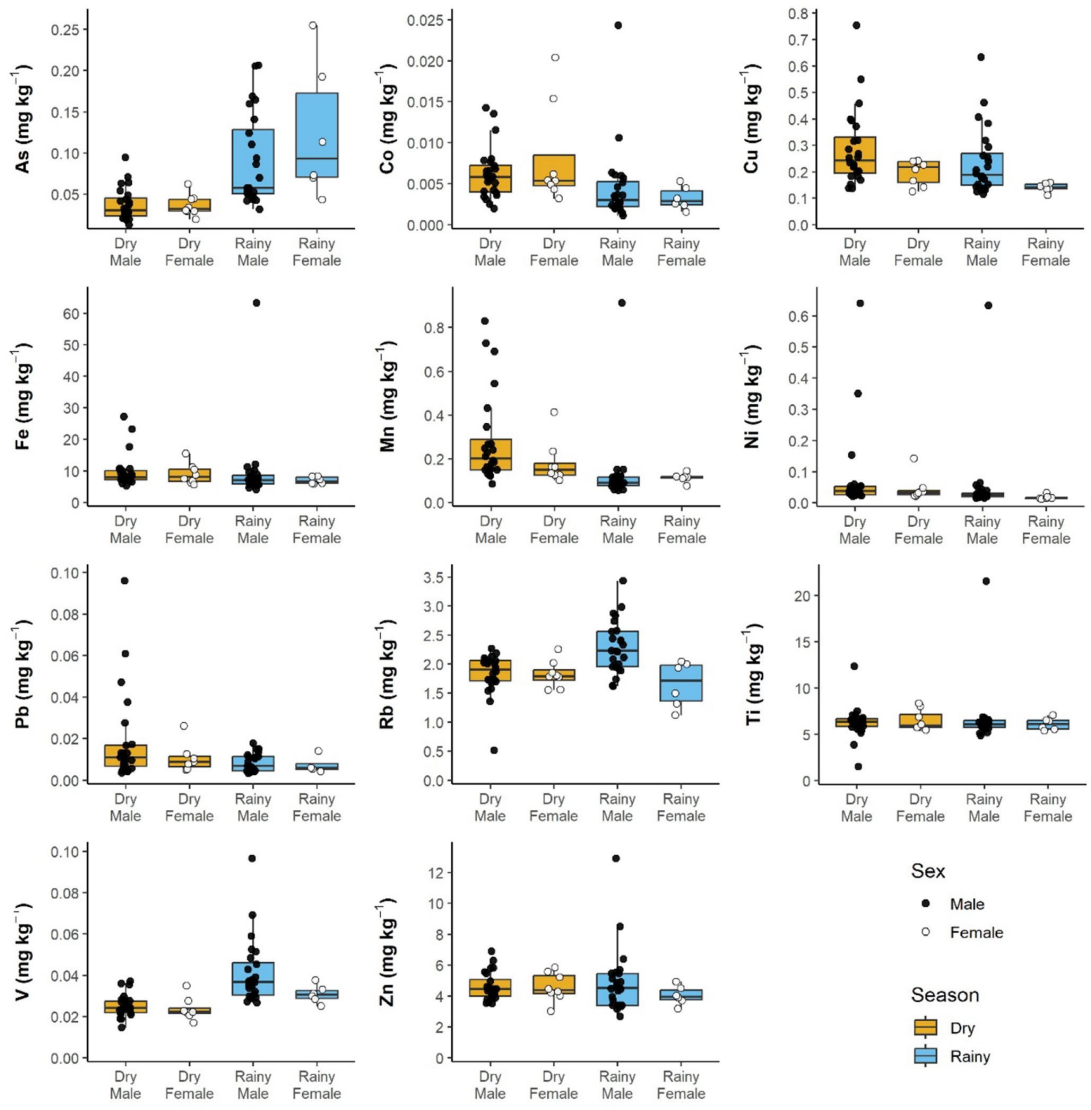
Concentrations of total metals in muscle of Nile tilapia (*Oreochromis niloticus*) from JLC, comparing seasons and separated by sex. Metals include arsenic (As), cobalt (Co), copper (Cu), iron (Fe), manganese (Mn), nickel (Ni), lead (Pb), rubidium (Rb), titanium (Ti), vanadium (V), and zinc (Zn). The central line is the median, the boxes span the interquartile range (IQR; Q1-Q3), whiskers extend to the most extreme observations within Q1 − 1.5×IQR and Q3 + 1.5×IQR; and points beyond these limits are shown as outliers (individual values). Colors indicate season (orange = dry; blue = rainy), and symbols denote sex (filled circles = males; open circles = females). Cadmium (Cd) and molybdenum (Mo) are not shown, as they were not consistently detected across seasons or sexes

The accumulation of contaminants in muscle tissue is influenced by various abiotic (water, sediment, and geographic location), biotic (size, sex, age, and reproduction stage), and ecological factors like growth rate, feeding sources, and trophic level [91–94]. Median values (mg kg⁻¹ w.w.) and distributions of total As, Co, Cu, Fe, Mn, Ni, Pb, Rb, Ti, V and Zn are described in Fig. 2. Mercury (Hg), although detected in lagoon water during the rainy season, was not detected in muscle tissue, likely reflecting its low bioavailability or limited incorporation into muscle under the studied conditions. No significant differences were observed between seasons or sexes (*p* > 0.05). Median concentrations of Fe in muscle were 324 mg kg⁻¹ (range: 117–400) in dry-season and 335 mg kg⁻¹ (range: 248–465) in rainy season, while Zn varied from 3.0 to 6.90 mg kg⁻¹ (dry) to 2.67–12.90 mg kg⁻¹ (rainy). Iron is generally reported at higher levels in most vertebrates as it is essential for several biological processes, such as oxygen transport and DNA synthesis; the same is noted for Zn, which participates in multiple metabolic pathways and enzymatic functions [95]. Although most concentrations were below commonly established guideline values, the focus of this study was on sublethal biological responses, which may occur even at low contaminant levels.

Among toxic elements, As was detected at 0.031 mg kg⁻¹ (0.013–0.095) in the dry season and 0.070 mg kg⁻¹ (0.032–0.255) in the rainy season; Pb, at 8.0 mg kg⁻¹ (dry) and 7.1 mg kg⁻¹ (rainy), ranging from 4.1 to 63.3 mg kg⁻¹. Although As is classified by the International Agency for Research on Cancer as carcinogenic to humans (Group 1), it should be noted that inorganic As is far more toxic than organic arsenic compounds [8, 11]. In fish, As occurs predominantly in organic forms, which is why many studies reports that dietary arsenic intake from fish does not pose a significant health risk to consumers [96], although inorganic As concentrations have been reported as ranging from 1 to 10% in fish and should not be ignored [97, 98].

The potentially toxic Ti reached 6.25 mg kg⁻¹ (1.51–12.40) in the dry season and 4.47 mg kg⁻¹ (2.67–12.90) in the rainy season. In recent years, the expanding use of titanium dioxide (TiO₂) in a wide range of consumer and industrial products has resulted in increased environmental release of Ti [99]. Major anthropogenic sources include sunscreens and personal care products, in which TiO₂ is widely used as a physical UV filter, as well as paints, plastics, construction materials, and wastewater effluents [99, 100]. These sources may contribute to elevated Ti levels in aquatic environments through direct release, urban runoff, and sediment accumulation [101].

The relatively homogeneous distribution of metals across groups is consistent with the spatial and temporal stability of contamination sources. As previously noted, the JLC has consistently exhibited chronic impacts, which likely exposes organisms to environmental contaminants on a long-term basis [15, 102, 103]. Similar patterns have been reported in other urbanized aquatic environments, where muscle tissue often shows low variability in total metal concentrations between seasons [104]. Moreover, subcellular fractionation shows that meaningful differences in metal bioaccumulation often appear only when examining intracellular compartments (e.g.: metallothionein-bound, organelle-associated) rather than total concentrations [90, 105].

#### Subcellular Metal Concentrations in Liver and Muscle

Subcellular metal and metalloid levels are presented in Table 3. No significant seasonal differences were observed in liver subcellular metal concentrations in either females or males. Likewise, no significant sex-related differences were detected within each sampling period. In muscle tissue, neither sex nor season had a significant effect on subcellular metal concentrations.

**Table 3:** Subcellular concentrations of metals and metalloids in liver and muscle of Nile tilapia from Jacarepaguá Lagoon Complex (JLC). Values are expressed as medians (mg kg⁻¹, w.w.) with range (minimum-maximum)

		Dry Season			Rainy Season		
	*Elements*	*Median*	*Min*	*Max*	*Median*	*Min*	*Max*
Liver	As	0.110	0.068	0.176	0.160	0.040	0.434
	Cd	0.004	0.001	0.009	0.004	0.001	0.029
	Co	0.048	0.028	0.120	0.042	0.018	0.633
	Cu	0.277	0.123	2.740	0.441	0.176	24.4
	Fe	48.4	25.9	61.5	47.3	17.0	233.0
	Hg	0.004	0.002	0.011	0.011	0.001	0.033
	Mg *	0.40	0.04	365	230	37	458.
	Mn	0.468	0.194	5.140	0.252	0.067	1.01
	Mo	0.031	0.018	0.046	0.044	0.005	0.354
	Ni	0.146	0.079	0.300	0.231	0.051	0.52
	Pb	0.031	0.003	0.208	0.035	0.006	0.074
	Rb	2.9	1.0	3.9	3.9	0.4	6.9
	Se	0.190	0.080	0.666	0.563	0.029	1.78
	Ti	5.4	2.5	7.7	5.8	1.1	13.2
	V	0.176	0.116	0.220	0.209	0.088	0.53
	Zn	7.9	6.1	21.1	12.3	6.1	29.2
Muscle	As	0.169	0.092	0.196	0.319	0.115	0.988
	Cd	0.005	0	0.009	0.001	0	0.007
	Co	0.02	0.001	0.386	0.007	0	0.036
	Cu	0.137	0.047	0.267	0.215	0.1	1.24
	Fe	10.2	3.03	41.2	6.25	1.01	27
	Hg	0.005	0.001	0.068	0.011	0.001	0.025
	Mg	0.33	0.04	814	421	286	604
	Mn	0.261	0.168	0.583	0.13	0.034	0.364
	Mo	0.023	0.001	0.055	0.025	0.003	0.393
	Ni	0.184	0.101	0.312	0.127	0.054	2.7
	Pb	0.026	0.01	0.149	0.017	0.005	0.076
	Rb	5.25	3.14	6.61	4.33	2.38	7.74
	Se	0.341	0.137	0.785	0.298	0.029	0.636
	Ti	7.66	5.52	10.8	6.03	4.02	8.3
	V	0.149	0.098	0.212	0.168	0.083	0.232
	Zn	11.4	3.38	20	5.72	0.89	11.9

*Asterisks denote significant differences (Kruskal–Wallis with Dunn’s post hoc, *p* < 0.05)

The lack of marked seasonal or sex-related variation, together with similar subcellular metal distributions in liver and muscle, suggests relatively stable exposure conditions. Although metal concentrations remained consistent across seasons, biomarker responses may still vary due to seasonal changes in environmental conditions and organism physiology, which can modulate metabolic activity, stress responses, and contaminant bioavailability. These patterns are more consistent with chronic, low-intensity metal inputs than with episodic contamination events, which typically result in pronounced temporal or tissue-specific variability [16, 106]. Although these results do not, by themselves, demonstrate the specific sources of contamination, they are consistent with previous evidence of continuous metal inputs to the Jacarepaguá Lagoon Complex, including inflowing rivers, domestic wastewater discharges, clandestine connections, and urban runoff ([107]; [108]; [109]).

### Human Health

Due to the absence of national regulatory limits for daily and weekly intake of metals and metalloids in Brazil, the Estimated Daily Intake (EDI) and Estimated Weekly Intake (EWI) values calculated for tilapia consumption were compared with international reference standards (FAO/WHO and European Union) (Tables S3 and S4 - Supplementary material). For human health risk assessment, mean metal concentrations in fish muscle were used to represent average exposure scenarios. In general, female consumer tends to be more susceptible to contaminant exposure due to lower body mass, while infants represent the most vulnerable group because of their developing metabolism and immune system, which reduces their capacity to metabolize or dilute toxicants [110, 111].

In the present study, As EDI values ranged from 0.000026 to 0.000146 mg kg ⁻¹ day⁻¹, and EWI from 0.000185 e 0.001019 mg kg ⁻¹ week⁻¹, with the lowest values observed in female children. Chronic exposure to As is linked to increased risks of cancer and other long-term diseases, such as skin disorders and cognitive impairment; [112]. Considering the FAO/WHO guideline of 0.003 mg kg⁻¹ day⁻¹, the values obtained herein did not exceed the recommended limit, indicating no apparent risk. Cadmium (Cd), known for its high toxicity and long biological half-life (10 to 30 years), is associated with carcinogenesis, osteoporosis, and renal impairment [113]. Cadmium EDI ranged from 0.000005 to 0.000010 mg kg⁻¹ day⁻¹ and EWI from 0.000033 to 0.000070 mg kg⁻¹ week⁻¹, remaining below the FAO/WHO limits (0.008 mg kg⁻¹ day⁻¹; 0.0058 mg kg⁻¹ week⁻¹). For Pb, a well-known affects the central nervous system in infants and may cause hypertension, renal and reproductive dysfunctions, and mutagenic effects in adults [114]. Although generally low in fish, Pb can accumulate in benthic organisms and cephalopods [114], the EDI ranged from 0.000006 to 0.000025 mg kg⁻¹ day⁻¹, and the EWI reached 0.000176 mg kg⁻¹ week⁻¹, raising concern for potential central nervous system impairment in sensitive groups. No reference or regulatory limits are currently available for Rb and Ti, precluding direct risk comparison.

Regarding essential elements, Cu and Fe are essential micronutrients involved in energy metabolism, immune function, oxygen transport, and connective-tissue integrity [82, 115]. However, excessive intake may lead to gastrointestinal distress, hepatic and renal damage, and neurological complications [116]. Mean Cu and Fe intakes were 0.00183 mg kg⁻¹ day⁻¹ and 0.07094 mg kg⁻¹ day⁻¹, respectively, both remaining well below the recommended limits of 0.005 mg kg⁻¹ day⁻¹ for Cu and 45 mg kg⁻¹ day⁻¹ for Fe (FAO/WHO). EDI and EWI values for Fe also fell within the tolerable upper intake level proposed for adults. Zinc, a cofactor for enzymes involved in protein synthesis, immunity, and neurological function [117], may also become toxic when excessively accumulated, causing nausea, vomiting, abdominal cramps, and diarrhea; chronic exposure can lead to anemia, pancreatic damage, and low HDL levels [118, 119]. EDI values for Zn ranged from 0.00328 to 0.00710 mg kg⁻¹ day⁻¹, and EWI from 0.02299 to 0.04970 mg kg⁻¹ week⁻¹, approaching but not exceeding the WHO/FAO upper limit of 0.040 mg kg⁻¹ day⁻¹ for adults. Although below the threshold, values near this range may still indicate potential for subclinical effects in susceptible populations.

The TCR calculations were performed only for As, Cd, and Pb, as only these have established oral cancer slope factors. These elements are known for their mutagenic, teratogenic, and carcinogenic potential in living organisms [113]. However, all TCR values (Tables S5 and S6, Supplementary Material) remained within the acceptable range even under the highest exposure frequency scenario [65, 120].

Target Hazard Quotients were calculated for consumption frequencies ranging from 1 to 5 days per week, estimated for As, Cu, Ni, V, and Zn, as only these have established oral reference doses (Tables S7–S11, Supplementary Material). All results were below the safety threshold of 1 across all age and sex groups, with no significant increase observed even at the highest weekly intake frequency. Although these values indicate no immediate risk, similar assessments in other regions have reported higher potential hazards. For instance, Hasanein et al., [121] evaluated THQ for metals such as Cd, Co, and Pb in tilapia *spp.* and catfish (*Clarias gariepinus*) from Lake Mariut (Egypt), finding THQ > 1 in liver and gill tissues, although muscle values remained < 1. Likewise, [122] reported THQ values exceeding 1 for Cd, Co, Pb, and Cr in liver, Co and Cr in gills, and Co in muscle of tilapia, indicating localized accumulation and potential tissue-specific health risks despite apparently safe concentrations in edible portions.

Finally, as toxic effects rarely occur in isolation, co-exposure may lead to synergistic or additive effects, where combined toxicity exceeds the sum of individual responses [123], the HI were calculated to evaluate the cumulative non-carcinogenic risk associated with simultaneous exposure to multiple metals through tilapia consumption (Tables S7–S11, Supplementary Material) [121, 124]. All estimated values remained below the threshold across age and sex groups, suggesting no significant non-carcinogenic health risk under current consumption scenarios.

### Fish Parameters

The GSI reflects reproductive investment and gonadal development, while the HSI is associated with liver metabolic activity, including energy storage, detoxification processes, and vitellogenin synthesis [125, 126]. The condition factor (K) provides an integrated measure of overall body condition and physiological well-being [127, 128]. Together, these indices offer complementary information on fish health and reproductive status and are sensitive indicators of physiological responses to environmental stressors, allowing the assessment of sublethal effects of pollutants on fish populations ([17, 129]; [130].

Organosomatic indexes are presented in Table 4. No significant differences between males and females were observed for the HSI or K in either sampling period. In contrast, the GSI exhibited clear sexual dimorphism, with consistently higher values in females than in males during the rainy season. The stability of HSI and K across seasons suggests that overall liver condition and body condition remained relatively constant during the study period [131, 132]. This pattern indicates that reproductive investment, as reflected by GSI, was not accompanied by detectable changes in liver size or general somatic condition, which may reflect physiological regulation that maintains somatic condition despite seasonal reproductive investment [133, 134].

**Table 4:** Organosomatic indexes of Nile tilapia (gonadosomatic index - GSI, hepatosomatic index - HSI, and Fulton’s condition factor -K). Stratification by sex was applied due to detected sex effects. Values are reported as medians (min–max)

	Dry Season				Rainy Season			
	Male		Female		Male		Female	
Indexes	Median	Min-max	Median	Min-max	Median	Min-max	Median	Min-max
HSI	0.2	0.07–0.8	0.13	0.07–0.4	0.2	0.07–0.6	0.1	0.04–0.3
GSI	0.4	0.03–0.6	1.5	0.5–3.9	0.7*	0.2–2.4	2.3*	0.3–3.9
K	1.9	1.6–1.9	1.7	1.6–2.2	1.7	1.5–2.0.5.0	1.8	1.6–2.3

*Asterisks indicate significant differences between sexes within the same season (Kruskal–Wallis with Dunn’s post hoc, *p* < 0.05)

### Biomarker Analysis

#### Antioxidant System and Oxidative Damage

**Fig. 3:**
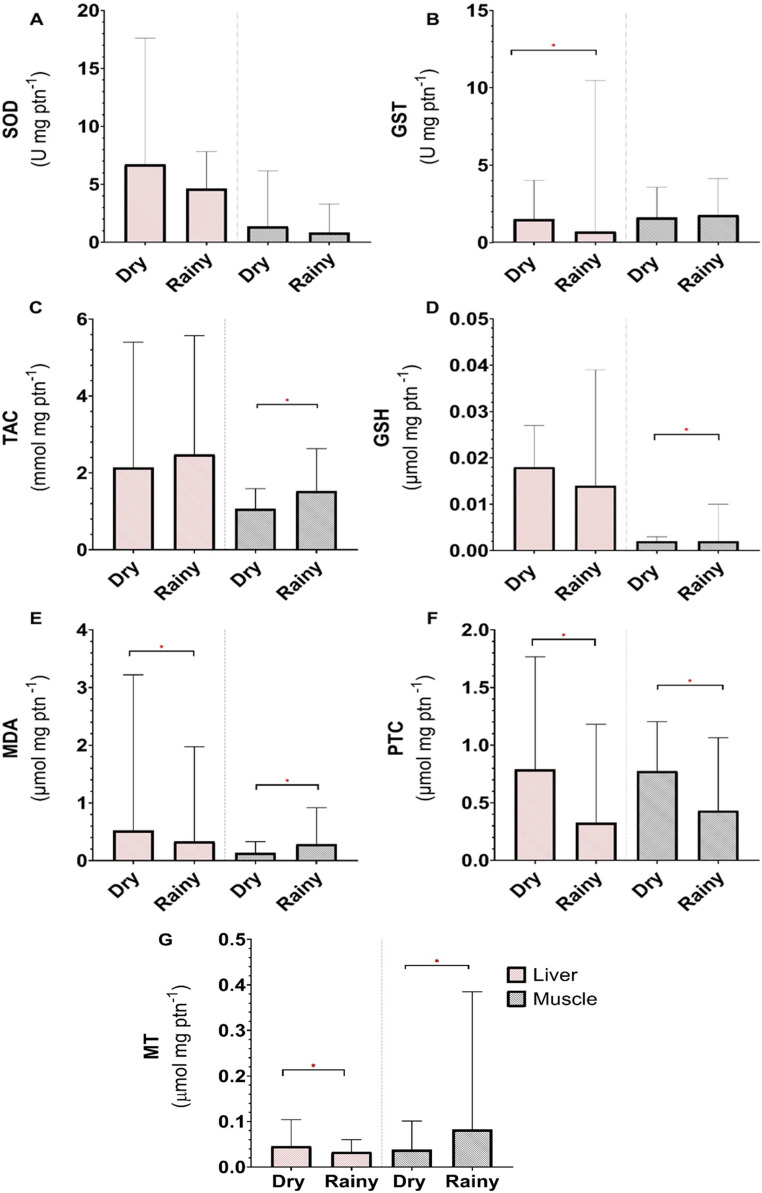
Descriptive statistics for antioxidant and oxidative damage biomarkers: (**A**) Superoxide dismutase (SOD), (**B**) glutathione s-transferase (GST), (**C**) total antioxidant capacity (TAC), (**D**) reduced glutathione (GSH), (**E**) malondialdehyde (MDA) as a marker of lipid peroxidation (LPO), (**F**) protein carbonylation (PTC) and (**G**) metallothionein (MT). * Asterisks indicate significant differences between seasons within the same tissue (Kruskal–Wallis with Dunn’s post hoc, *p* < 0.05)

Antioxidant and oxidative damage biomarkers results are depicted in Fig. 3. Concerning liver, SOD (Fig. 3A) increased 44.8% during the dry season compared to rainy season albeit non-significantly. GST (Fig. 3B) increased significantly 110.2% during the dry season, while GSH (Fig. 3D) remained similar, with no significant change. Metallothionein (Fig. 3G) was significantly higher during the dry period, increasing 37.3%. Oxidative damage biomarkers also peaked in the dry season, in which MDA (Fig. 3E) increased 56.4% and PTC (Fig. 3F), 141.6%, both significant when compared to the rainy season. During the rainy season, GSH (+ 20.8%) and TAC (+ 16.4%) (Fig. 3C) were higher, although non-significantly.

In muscle tissue, most biomarkers were higher in the rainy season, as follows: GST (Fig. 3B) showed a modest increase of 9.8%, while MT (*p* > 0.05) (Fig. 3G) increased significantly by 117.9%. TAC showed a significant increase of 43% compared to the dry period (1.53 mmol mg⁻¹ ptn, 0.600–2.63) (Fig. 3C). GSH (Fig. 3D) remained similar during both seasons. Conversely, SOD (Fig. 3A) activity was higher in the dry season (+ 66.3%), although this difference was non-significant. Concerning oxidative damage, MDA (Fig. 3E) and PTC (Fig. 3F) were both significantly higher in the rainy season (+ 114.2% and 80.6%; 0.78 µmol mg⁻¹ ptn, 0.425–1.20.425.20, respectively,

During the dry season, reduced dilution and increased physicochemical variability may contribute to enhanced oxidative stress in fish [135]. Superoxide dismutase (SOD), a key antioxidant enzyme involved in the dismutation of superoxide radicals (converting O₂•⁻ into H₂O₂ [136], showed increased activity in both liver and muscle tissues. Although differences were not statistically significant, metal concentrations were numerically higher in muscle and in hepatic and muscular subcellular fractions during this period. This pattern suggests a potential increase in reactive oxygen species production and activation of antioxidant defenses. If these responses are not sufficient to counterbalance oxidative processes, the accumulation of reactive intermediates may result in cellular damage, as indicated by the higher levels of protein carbonylation (PTC) observed in the dry season [137, 138]. Overall, these findings are consistent with increased oxidative pressure under dry-season conditions. However, the relationship between biomarker responses and environmental drivers, including metal exposure, should be interpreted with caution, as causal mechanisms were not directly assessed in this study.

The liver is the main biotransformation organ in most vertebrates [139]. Metallothionein in this organ sequesters metals and reduces oxidative stress via metal sequestration/chelation [128, 140]; Glutathione S-transferase (GST) catalyzes GSH conjugation for phase-II detoxification [141]; GSH is the main intracellular non-enzymatic antioxidant [142]; Total antioxidant capacity (TAC) reflects the sum of enzymatic and non-enzymatic antioxidant defenses [142]. Thus, hepatic peaks of MT, GST, and TAC, accompanied by higher SOD and PTC in the dry season, are consistent with greater oxidative pressure under lower dilution rates. Malondialdehyde exhibited the same trend, reinforcing this hypothesis and suggesting that antioxidant capacity may have been insufficient to fully prevent damage [10].

In the rainy season, the opposite pattern is observed, with peaks of MT, TAC, GST, and MDA (LPO) in muscle. This profile is consistent with greater food supply and urban runoff, which intensify the formation of lipid hydroperoxides and other organic electrophiles in muscle [5, 12]. In this situation, GST (detoxifies electrophilic compounds) and the non-enzymatic components captured by TAC (reflects total antioxidant availability) tend to respond more directly [141, 143, 144], without requiring a proportional increase in SOD. In addition, muscular MT may increase in parallel with dietary metal input [138, 145].

#### Vitellogenin (VTG)

**Fig. 4:**
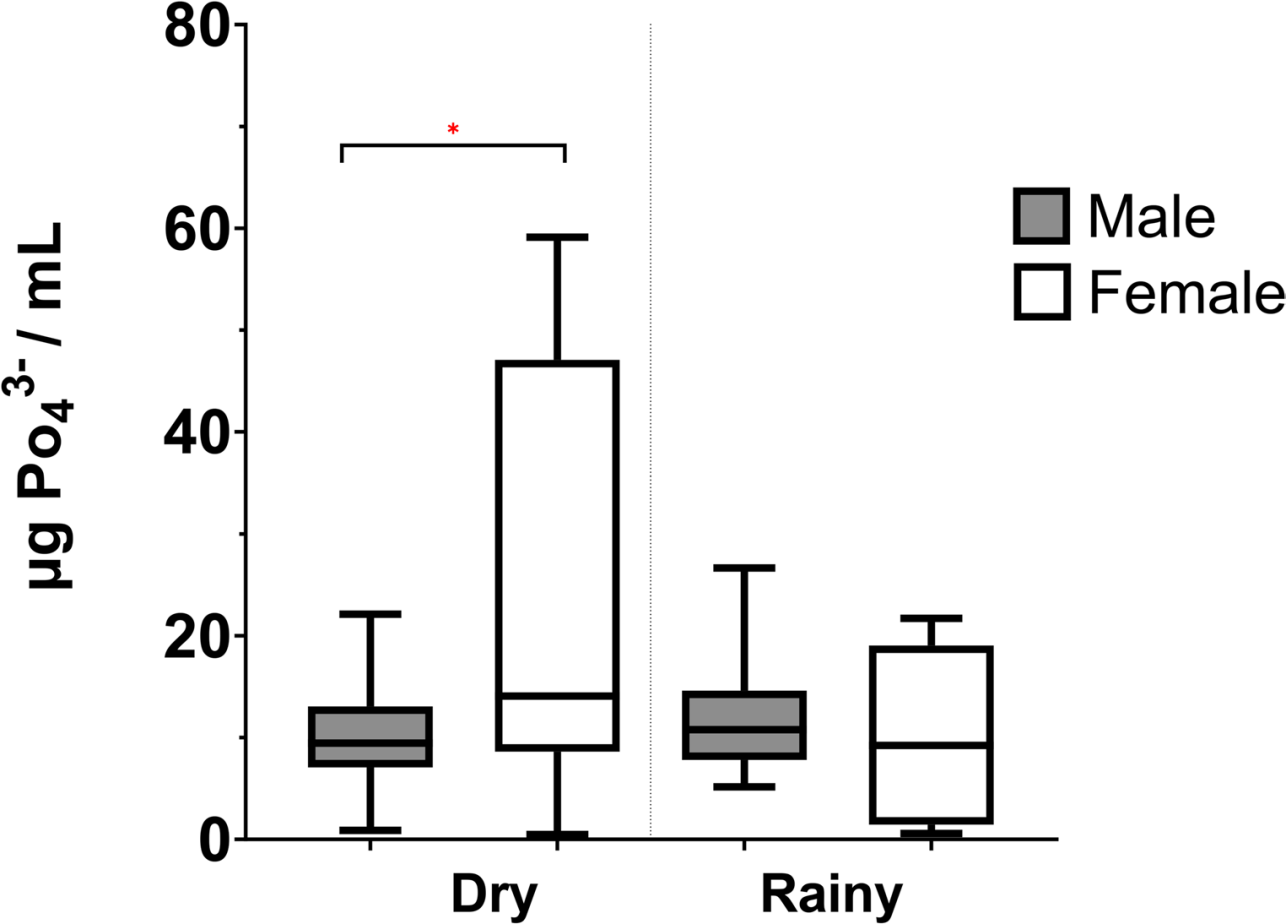
Alkaline-labile phosphate (ALP) in *Oreochromis niloticus* by season and sex. Concentrations are expressed as µg PO₄³⁻ mL⁻¹ plasma. The central line is the median, the boxes span the interquartile (IQR; Q1-Q3), whiskers extend to the most extreme observations within Q1 − 1.5×IQR and Q3 + 1.5×IQR; and points beyond these limits are shown as outliers (individual values). * Indicate significant differences between sex (Kruskal–Wallis with Dunn’s post hoc, *p* < 0.05)

Plasma vitellogenin levels are presented in Fig. 4. During the dry season, females showed higher VTG than males (14.10 vs. 9.45 µg PO₄³⁻ mL⁻¹); with a male/female ratio (M/F) = 0.67 (*p* < 0.05), as expected based on the estrogen-dependent regulation of vitellogenin synthesis in females [146]. In contrast, no significant sex-related difference was observed during the rainy season, although males exhibited slightly higher median VTG levels than females (11.10 vs. 9.23 µg PO₄³⁻ mL⁻¹; M/F = 1.20). Overall, VTG showed a seasonal shift in its sex-related pattern, with males presenting 33% lower VTG levels than females in the dry period and approximately 20% higher levels during the rainy season, suggesting a possible relative increase of VTG in males under wet-season conditions.

Previous studies have reported seasonal variations in VTG in fish [147–150]. In tropical/subtropical regions of South America, VTG activity is almost continuous when water remains warm (≥ 23–24 °C), with fractional spawning and short reproductive cycles (21–24 days) [151]. Accordingly, phosphoproteins tend to increase in female during vitellogenesis, with mineral adjustments for yolk formation [152, 153].

In the present study, the absence of clear sex-related differences in VTG during the rainy season, combined with slightly higher median values in males, may indicate a seasonal shift in VTG patterns. Although environmental inputs associated with rainfall, such as increased runoff, could influence this response [14, 154, 155], these factors were not directly measured. Therefore, the observed pattern should be interpreted with caution. Similar trends have been reported in urban aquatic systems, where elevated VTG levels in male fish have been associated with environmental exposure, although underlying mechanisms remain context-dependent [148, 156, 157].

### Principal Components Analysis (PCA)

**Fig. 5:**
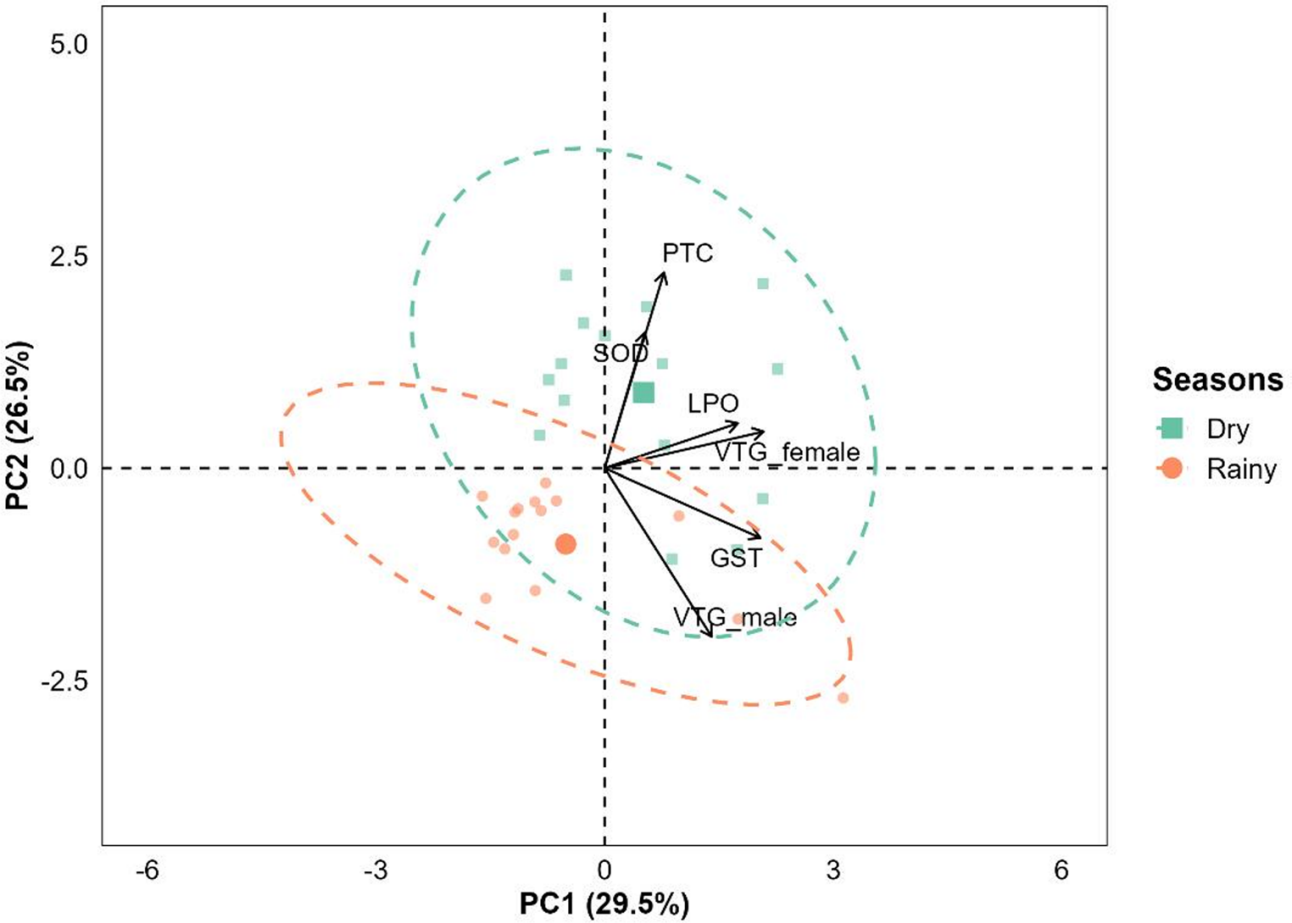
Principal Component Analysis (PCA) of liver biomarkers in Nile tilapia (*Oreochromis niloticus*) from Jacarepaguá Lagoon Complex (JLC) the antioxidant system, including superoxide dismutase (SOD) glutathione-S-transferase (GST), lipid peroxidation (LPO), protein carbonyls (PTC) and vitellogenin (Vtg) from female and male fish, across dry (green squares) and rainy (orange circles) seasons. The variables were explained by PC1 (29.5%) and PC2 (26.5%) factors. Bartlett p-value < 0.05. KMO = 0.53

A PCA revealed patterns consistent with multivariate stress responses (Fig. 5). PCA was restricted to liver samples (Bartlett’s test *p* < 0.05), with exclusion of the muscle samples due to inadequate correlation (*p* > 0.05). The first two components explained 56% of the total variance (PC1: 29.5%; PC2: 26.5%). According loading values (Table S12): PC1, driven mainly by female VTG, GST and LPO (with secondary contributions from male VTG and PTC), suggests an axis that linking endocrine response and phase-II detoxification with lipid damage. Concerning this interpretation, the increase in GST did not seem sufficient to contain ROS generated, with protein and lipid damage emerging in the same direction, consistent with moderate correlations among these variables (Table S13). In contrast, PC2 was dominated by PTC and SOD (positive) and male VTG (negative), contrasting oxidative damage/antioxidant activity with male VTG, capturing a gradient of oxidative stress in which primary antioxidant response and protein damage tend to increase together, while the male VTG signal projects in the opposite direction. The contrasts oxidative damage/antioxidant were consistent with peroxide pressure associated with SOD that was insufficient to prevent protein oxidation [158].Seasonal differences were observed, with fish sampled in the dry season clustered in positive PC1, consistent with endocrine activation, phase II detoxification and lipid damage. In the rainy period, fish were positioned predominantly in positive PC2, suggesting greater protein damage accompanied by an antioxidant response. Similar to studies that relate multiple stressors to tissue responses, these results are compatible with distinct exposure profiles modulated by hydrology and seasonality [13, 159]. These results suggest the multi-stressor character of the JLC and suggest that hydrological dynamics modulate contaminant bioavailability, directing divergent ecotoxicological pathways in Nile tilapia.

## Conclusion

The JLC is under chronic and diffuse anthropogenic pressure, by a poor water-quality index and by biological wild tilapia responses. Metal monitoring confirmed the environmental availability of several elements (e.g. As, Fe, Mn, Rb and V) in surface waters. Despite modest seasonal differences in metal accumulation, the presence of multiple metals in hepatic and muscular subcellular fractions confirms that tilapias are exposed and internalize these contaminants.

The determined biomarkers revealed clear physiological effects. The dry season imposed greater hepatic oxidative challenge, with increased SOD was not sufficient to prevent protein oxidation (increased PTC). In contrast, the rainy season elicited antioxidant mobilization in muscle, with increases in GST, TAC, and MT, consistent with urban drainage and input of contaminants. Additionally, VTG induction in males during the rainy season can indicates endocrine modulation, suggesting the potential influence of urban runoff and endocrine-active substances in the system. These findings demonstrate that sublethal oxidative and endocrine disturbances occur even under diffuse and chronic contamination, revealing early biological impairment that would go unnoticed under conventional monitoring restricted to isolated approach. This reinforces the value of multibiomarker studies as a sensitive tool for detecting biological effects of contamination in urban coastal lagoons.

Human health risk assessments indicate tilapia from the JLC does not pose significant non-carcinogenic or carcinogenic risks, with all EDI, EWI, THQ, TCR, and HI values remaining below internationally accepted thresholds. However, the proximity of some estimates to upper intake limits, especially for vulnerable groups such as infants, highlights the need for continued monitoring and supports the inclusion of fish-consumption risk evaluation in routine coastal-lagoon management.

In conclusion, JLC degradation is not a local case but reflects a recurrent pattern in eutrophic and densely urbanized lagoon systems, with implications for environmental management and public health. Given the scarcity of studies with this dynamic and multidisciplinary focus in the region, it is recommended to incorporate sediment assessments, speciation of metals, expand investigations with other endocrine and oxidative effect biomarkers to better assess the impact of metal contamination in coastal environments.

## Supplementary Information

Below is the link to the electronic supplementary material.

Supplementary Material 1 (DOCX 136 KB)

## Data Availability

No datasets were generated or analysed during the current study.
