# Supplementary material for: Metal-Driven Biochemical and Physiological Stress in Fish Inhabiting an Urban Coastal Lagoon: Evidence from an Integrated Multibiomarker Approach
Source: Biol Trace Elem Res. 2026 May 16;204(9):6678–99. doi: 10.1007/s12011-026-05140-3 (PMC13437577; doi:10.1007/s12011-026-05140-3)
Supplement: Supplementary file 1 [file 12011_2026_5140_MOESM1_ESM.docx]

**Supplementary Material**

**Metal-driven biochemical and physiological stress in fish inhabiting an urban coastal lagoon: evidence from an integrated multibiomarker approach**

Julia Araujo Alves^1^, Priscila M. O. Muniz Cunha^2^, Guilherme de Souza Meireles^1^, Fábio Veríssimo Correia^3^; André Luís de Sá Salomão^2^; Rachel Ann Hauser-Davis ^1^, Enrico Mendes Saggioro^1^

^1^ Environmental Health Assessment and Promotion Laboratory (LAPSA), Oswaldo Cruz Institute (IOC), Oswaldo Cruz Foundation (Fiocruz), Rio de Janeiro, Brazil.

^2^Bioremediation and Phytotechnology Laboratory (LABIFI), Department of Environmental Engineering, Rio de Janeiro State University (UERJ), Rio de Janeiro, Brazil.

^3^Environmental Health Laboratory, Department of Natural Sciences, Federal University of the State of Rio de Janeiro (UNIRIO), Rio de Janeiro, Brazil.

*Corresponding author:

E-mail: enrico.saggioro@fiocruz.br (Enrico M Saggioro)

Postal address: Environmental Health Evaluation and Promotion Laboratory, Oswaldo Cruz Institute, Oswaldo Cruz Foundation, 4365 Brasil Avenue, Manguinhos, Rio de Janeiro, RJ, Brazil.

**Table S1:** Observed and certified values (mg kg^-1^) and recovery percentages for the Certified Reference Materials: DORM-5, National Research Council Canada; NIST SRM 2976, National Institute of Standards and Technology, USA; BCR 668 (Mussel Tissue), from the European Commission.

|  | BCR 668 | | | DORM-5 | | | NIST 2976 | | |
| --- | --- | --- | --- | --- | --- | --- | --- | --- | --- |
| Elements | Observed | Certified | Recovery | Observed | Certified | Recovery | Observed | Certified | Recovery |
| As | 6.3 | 7.1 | 89.5 | 14.4 | 13.3 | 108.9 | 11.8 | 13.3 | 88.7 |
| Co |  |  |  | 0.04 | 0.06 | 71.54 |  |  |  |
| Cd | 0.238 | 0.275 | 86.379 | 0.167 | 0.148 | 112.883 | 0.717 | 0.820 | 87.411 |
| Cu |  |  |  | 2.79 | 3.30 | 84.71 | 3.62 | 4.02 | 90.25 |
| Fe |  |  |  | 79.34 | 113.00 | 70.21 | 150.02 | 171.00 | 87.73 |
| Hg |  |  |  | 0.35 | 0.31 | 111.49 | 0.06 | 0.06 | 99.19 |
| Mn |  |  |  | 0.76 | 1.06 | 71.65 |  |  |  |
| Mo | 1.55 | 1.99 | 78.16 | 0.12 | 0.13 | 92.42 |  |  |  |
| Ni |  |  |  | 0.35 | 0.44 | 80.56 | 0.67 | 0.93 | 72.37 |
| Pb |  |  |  | 0.06 | 0.05 | 111.34 | 1.03 | 1.19 | 86.58 |
| Rb |  |  |  | 2.19 | 2.76 | 79.53 |  |  |  |
| Se |  |  |  | 2.91 | 2.40 | 121.26 | 2.43 | 1.80 | 135.23 |
| V |  |  |  | 0.396 | 0.347 | 114.002 |  |  |  |
| Zn | 62.37 | 70.70 | 88.22 | 33.77 | 28.70 | 117.67 | 129.21 | 137.00 | 94.32 |

**Table S2**: Limits of Detection (LOD) and Quantification (LOQ) for metals and metalloids in water (mg L⁻¹) and fish tissue (mg kg^-1^ wet weight). Fish tissues values refer to total and subcellular fractions. Calculations followed INMETRO (2016).

|  | LOD | | | LOQ | | |
| --- | --- | --- | --- | --- | --- | --- |
| Elements | Water  (mg L^–1^) | Total  (mg kg^–1^) | Subcell  (mg kg^–1^) | Water  (mg L^–1^) | Total  (mg kg^–1^) | Subcell  (mg kg^–1^) |
| As | 0.01 | 0.002 | 0.004 | 0.03 | 0.007 | 0.014 |
| Cd | 0.001 | 0.001 | 0.001 | 0.003 | 0.002 | 0.001 |
| Co | 0.002 | 0.001 | 0.001 | 0.006 | 0.001 | 0.001 |
| Cu | 0.004 | 0.001 | 0.012 | 0.013 | 0.001 | 0.040 |
| Fe | 0.24 | 0.90 | 1.049 | 0.80 | 3.00 | 3.500 |
| Hg | 0.003 | 0.002 | 0.002 | 0.010 | 0.010 | 0.008 |
| Mg | 0.03 | 0.05 | 0.057 | 0.10 | 0.17 | 0.190 |
| Mn | 0.003 | 0.01 | 0.002 | 0.010 | 0.02 | 0.010 |
| Mo | 0.01 | 0.001 | 0.009 | 0.02 | 0.001 | 0.030 |
| Ni | 0.01 | 0.002 | 0.007 | 0.03 | 0.007 | 0.024 |
| Pb | 0.002 | 0.001 | 0.003 | 0.006 | 0.003 | 0.009 |
| Rb | 0.001 | 0.001 | 0.001 | 0.002 | 0.002 | 0.001 |
| Se | 0.06 | 0.07 | 0.076 | 0.20 | 0.20 | 0.250 |
| Ti | 0.02 | 0.05 | 0.009 | 0.07 | 0.17 | 0.030 |
| V | 0.01 | 0.001 | 0.002 | 0.04 | 0.001 | 0.007 |
| Zn | 0.01 | 0.01 | 0.122 | 0.05 | 0.02 | 0.405 |

**Table S3:** Estimated Daily Intake (EDI) (mean ± SD), expressed in mg kg ⁻¹ day⁻¹, calculations concerning Oreochromis niloticus consumption by adult, child, and infant males and females during the dry, rainy, and mixed seasons.

|  | **Group** | **Sex** | **As** | **Cd** | **Co** | **Cu** | **Fe** | **Mg** | **Mn** | **Mo** | **Ni** | **Pb** | **Rb** | **Ti** | **V** | **Zn** |
| --- | --- | --- | --- | --- | --- | --- | --- | --- | --- | --- | --- | --- | --- | --- | --- | --- |
| **EDI Rainy Season** | Babies | Males | 0,001E-04 ± 8,418E-08 | NA | 0,580E-08 ± 0,585E-08 | 0,003E-04 ± 0,002E-04 | 0,124E-04 ± 0,142E-04 | 4,516E-04 ± 0,616E-04 | 0,002E-04 ± 0,002E-04 | 0,748E-08 ± 0,334E-08 | 6,321E-08 ± 0,002E-04 | 1,135E-08 ± 0,579E-08 | 0,030E-04 ± 0,007E-04 | 0,090E-04 ± 0,039E-04 | 5,346E-08 ± 2,033E-08 | 0,065E-04 ± 0,026E-04 |
|  |  | Females | 0,001E-04 ± 9,260E-08 | NA | 0,638E-08 ± 0,643E-08 | 0,003E-04 ± 0,002E-04 | 0,136E-04 ± 0,156E-04 | 4,968E-04 ± 0,677E-04 | 0,002E-04 ± 0,002E-04 | 0,823E-08 ± 0,368E-08 | 6,953E-08 ± 0,002E-04 | 1,248E-08 ± 0,637E-08 | 0,033E-04 ± 0,008E-04 | 0,098E-04 ± 0,043E-04 | 5,881E-08 ± 2,236E-08 | 0,071E-04 ± 0,029E-04 |
|  | Children | Males | 0,001E-04 ± 6,431E-08 | NA | 0,443E-08 ± 0,447E-08 | 0,002E-04 ± 0,001E-04 | 0,095E-04 ± 0,108E-04 | 3,450E-04 ± 0,470E-04 | 0,001E-04 ± 0,002E-04 | 0,571E-08 ± 0,255E-08 | 4,828E-08 ± 0,001E-04 | 0,867E-08 ± 0,442E-08 | 0,023E-04 ± 0,005E-04 | 0,068E-04 ± 0,030E-04 | 4,084E-08 ± 1,553E-08 | 0,049E-04 ± 0,020E-04 |
|  |  | Females | 0,001E-04 ± 7,015E-08 | NA | 0,483E-08 ± 0,487E-08 | 0,002E-04 ± 0,001E-04 | 0,103E-04 ± 0,118E-04 | 3,764E-04 ± 0,513E-04 | 0,001E-04 ± 0,002E-04 | 0,623E-08 ± 0,279E-08 | 5,267E-08 ± 0,001E-04 | 0,945E-08 ± 0,483E-08 | 0,025E-04 ± 0,006E-04 | 0,075E-04 ± 0,033E-04 | 4,455E-08 ± 1,694E-08 | 0,054E-04 ± 0,022E-04 |
|  | Adults | Males | 6,935E-08 ± 4,410E-08 | NA | 0,304E-08 ± 0,306E-08 | 0,002E-04 ± 8,525E-08 | 0,065E-04 ± 0,074E-04 | 2,366E-04 ± 0,323E-04 | 8,917E-08 ± 0,001E-04 | 0,392E-08 ± 0,175E-08 | 3,311E-08 ± 7,969E-08 | 0,594E-08 ± 0,303E-08 | 0,015E-04 ± 0,004E-04 | 0,047E-04 ± 0,021E-04 | 2,800E-08 ± 1,065E-08 | 0,034E-04 ± 0,014E-04 |
|  |  | Females | 7,281E-08 ± 4,630E-08 | NA | 0,319E-08 ± 0,322E-08 | 0,002E-04 ± 8,951E-08 | 0,068E-04 ± 0,078E-04 | 2,484E-04 ± 0,339E-04 | 9,363E-08 ± 0,001E-04 | 0,411E-08 ± 0,184E-08 | 3,476E-08 ± 8,368E-08 | 0,624E-08 ± 0,319E-08 | 0,016E-04 ± 0,004E-04 | 0,049E-04 ± 0,022E-04 | 2,940E-08 ± 1,118E-08 | 0,035E-04 ± 0,015E-04 |
| **EDI Dry Season** | Babies | Males | 5,049E-08 ± 2,496E-08 | 0,905E-08 ± 0,505E-08 | 0,917E-08 ± 0,559E-08 | 0,004E-04 ± 0,002E-04 | 0,131E-04 ± 0,066E-04 | 4,338E-04 ± 0,733E-04 | 0,004E-04 ± 0,003E-04 | 4,667E-08 ± 0,001E-04 | 9,728E-08 ± 0,002E-04 | 2,291E-08 ± 2,688E-08 | 0,025E-04 ± 0,005E-04 | 0,087E-04 ± 0,022E-04 | 3,388E-08 ± 0,732E-08 | 0,063E-04 ± 0,012E-04 |
|  |  | Females | 5,554E-08 ± 2,746E-08 | 0,995E-08 ± 0,556E-08 | 1,009E-08 ± 0,615E-08 | 0,004E-04 ± 0,002E-04 | 0,144E-04 ± 0,073E-04 | 4,772E-04 ± 0,807E-04 | 0,004E-04 ± 0,003E-04 | 5,133E-08 ± 0,001E-04 | 0,001E-04 ± 0,002E-04 | 2,520E-08 ± 2,957E-08 | 0,027E-04 ± 0,005E-04 | 0,095E-04 ± 0,024E-04 | 3,726E-08 ± 0,805E-08 | 0,069E-04 ± 0,014E-04 |
|  | Children | Males | 3,857E-08 ± 1,907E-08 | 0,691E-08 ± 0,386E-08 | 0,701E-08 ± 0,427E-08 | 0,003E-04 ± 0,001E-04 | 0,100E-04 ± 0,051E-04 | 3,314E-04 ± 0,560E-04 | 0,003E-04 ± 0,002E-04 | 3,565E-08 ± 8,042E-08 | 7,431E-08 ± 0,001E-04 | 1,750E-08 ± 2,053E-08 | 0,019E-04 ± 0,003E-04 | 0,066E-04 ± 0,017E-04 | 2,588E-08 ± 0,559E-08 | 0,048E-04 ± 0,009E-04 |
|  |  | Females | 4,208E-08 ± 2,080E-08 | 0,754E-08 ± 0,421E-08 | 0,764E-08 ± 0,466E-08 | 0,003E-04 ± 0,002E-04 | 0,109E-04 ± 0,055E-04 | 3,615E-04 ± 0,611E-04 | 0,003E-04 ± 0,002E-04 | 3,889E-08 ± 8,773E-08 | 8,107E-08 ± 0,001E-04 | 1,909E-08 ± 2,240E-08 | 0,021E-04 ± 0,004E-04 | 0,072E-04 ± 0,019E-04 | 2,823E-08 ± 0,610E-08 | 0,052E-04 ± 0,010E-04 |
|  | Adults | Males | 2,645E-08 ± 1,308E-08 | 0,474E-08 ± 0,265E-08 | 0,480E-08 ± 0,293E-08 | 0,002E-04 ± 9,444E-08 | 0,069E-04 ± 0,035E-04 | 2,272E-04 ± 0,384E-04 | 0,002E-04 ± 0,001E-04 | 2,444E-08 ± 5,514E-08 | 5,096E-08 ± 8,644E-08 | 1,200E-08 ± 1,408E-08 | 0,013E-04 ± 0,002E-04 | 0,045E-04 ± 0,012E-04 | 1,774E-08 ± 0,383E-08 | 0,033E-04 ± 0,006E-04 |
|  |  | Females | 2,777E-08 ± 1,373E-08 | 0,498E-08 ± 0,278E-08 | 0,504E-08 ± 0,308E-08 | 0,002E-04 ± 9,916E-08 | 0,072E-04 ± 0,037E-04 | 2,386E-04 ± 0,403E-04 | 0,002E-04 ± 0,001E-04 | 2,567E-08 ± 5,790E-08 | 5,350E-08 ± 9,076E-08 | 1,260E-08 ± 1,478E-08 | 0,014E-04 ± 0,002E-04 | 0,048E-04 ± 0,012E-04 | 1,863E-08 ± 0,402E-08 | 0,034E-04 ± 0,007E-04 |
| **EDI Rainy and Dry Season** | Babies | Males | 9.012E-05 ± 7.341E-05 | 9.049E-06 ± 5.052E-06 | 7.541E-06 ± 5.918E-06 | 3.294E-04 ± 1.737E-04 | 1.275E-02 ± 1.087E-02 | 4.424E-01 ± 6.795E-02 | 2.638E-04 ± 2.511E-04 | 2.427E-05 ± 6.836E-05 | 8.079E-05 ± 1.585E-04 | 1.753E-05 ± 2.072E-05 | 2.718E-03 ± 6.215E-04 | 8.802E-03 ± 3.148E-03 | 4.335E-05 ± 1.792E-05 | 6.359E-03 ± 2.027E-03 |
|  |  | Females | 9.913E-05 ± 8.075E-05 | 9.954E-06 ± 5.557E-06 | 8.295E-06 ± 6.510E-06 | 3.624E-04 ± 1.911E-04 | 1.403E-02 ± 1.196E-02 | 4.867E-01 ± 7.474E-02 | 2.902E-04 ± 2.762E-04 | 2.670E-05 ± 7.520E-05 | 8.887E-05 ± 1.744E-04 | 1.928E-05 ± 2.279E-05 | 2.989E-03 ± 6.837E-04 | 9.682E-03 ± 3.463E-03 | 4.769E-05 ± 1.971E-05 | 6.995E-03 ± 2.229E-03 |
|  | Children | Males | 6.884E-05 ± 5.607E-05 | 6.913E-06 ± 3.859E-06 | 5.761E-06 ± 4.521E-06 | 2.517E-04 ± 1.327E-04 | 9.741E-03 ± 8.307E-03 | 3.380E-01 ± 5.190E-02 | 2.015E-04 ± 1.918E-04 | 1.854E-05 ± 5.222E-05 | 6.172E-05 ± 1.211E-04 | 1.339E-05 ± 1.583E-05 | 2.076E-03 ± 4.748E-04 | 6.724E-03 ± 2.405E-03 | 3.312E-05 ± 1.369E-05 | 4.857E-03 ± 1.548E-03 |
|  |  | Females | 7.510E-05 ± 6.117E-05 | 7.541E-06 ± 4.210E-06 | 6.284E-06 ± 4.932E-06 | 2.745E-04 ± 1.448E-04 | 1.063E-02 ± 9.062E-03 | 3.687E-01 ± 5.662E-02 | 2.199E-04 ± 2.093E-04 | 2.023E-05 ± 5.697E-05 | 6.733E-05 ± 1.321E-04 | 1.461E-05 ± 1.727E-05 | 2.265E-03 ± 5.179E-04 | 7.335E-03 ± 2.624E-03 | 3.613E-05 ± 1.493E-05 | 5.299E-03 ± 1.689E-03 |
|  | Adults | Males | 4.720E-05 ± 3.845E-05 | 4.740E-06 ± 2.646E-06 | 3.950E-06 ± 3.100E-06 | 1.726E-04 ± 9.101E-05 | 6.679E-03 ± 5.696E-03 | 2.318E-01 ± 3.559E-02 | 1.382E-04 ± 1.315E-04 | 1.271E-05 ± 3.581E-05 | 4.232E-05 ± 8.305E-05 | 9.180E-06 ± 1.085E-05 | 1.424E-03 ± 3.256E-04 | 4.610E-03 ± 1.649E-03 | 2.271E-05 ± 9.385E-06 | 3.331E-03 ± 1.062E-03 |
|  |  | Females | 4.957E-05 ± 4.037E-05 | 4.977E-06 ± 2.779E-06 | 4.148E-06 ± 3.255E-06 | 1.812E-04 ± 9.556E-05 | 7.013E-03 ± 5.981E-03 | 2.433E-01 ± 3.737E-02 | 1.451E-04 ± 1.381E-04 | 1.335E-05 ± 3.760E-05 | 4.444E-05 ± 8.720E-05 | 9.639E-06 ± 1.140E-05 | 1.495E-03 ± 3.418E-04 | 4.841E-03 ± 1.732E-03 | 2.384E-05 ± 9.854E-06 | 3.497E-03 ± 1.115E-03 |
| * EFSA, (2006);FAO/ WHO, (1995);USEPA, (2000); Cu: Babies (<1 mg kg⁻¹); Children (<3 mg kg⁻¹); Adults (<5 mg kg⁻¹); Zn: Babies (<7 mg kg⁻¹); Children (<22 mg kg⁻¹); Adults (<25 mg kg⁻¹) . | | | | | | | | | | | | | | | | |

**Table S4:** Estimated Weekly Intake (EWI) (mean ± SD) calculations concerning Oreochromis niloticus consumption by adult, child, and infant males and females during the dry, rainy, and mixed seasons.

|  | **Group** | **Sex** | **As** | **Cd** | **Co** | **Cu** | **Fe** | **Mg** | **Mn** | **Mo** | **Ni** | **Pb** | **Rb** | **Ti** | **V** | **Zn** |
| --- | --- | --- | --- | --- | --- | --- | --- | --- | --- | --- | --- | --- | --- | --- | --- | --- |
| **EWI Rainy Season** | Babies | Males | 0,009E-04 ± 0,006E-04 | NA | 4,061E-08 ± 4,092E-08 | 0,021E-04 ± 0,011E-04 | 0,867E-04 ± 0,993E-04 | 0,003E+00 ± 4,311E-04 | 0,012E-04 ± 0,014E-04 | 5,234E-08 ± 2,341E-08 | 0,004E-04 ± 0,011E-04 | 7,942E-08 ± 4,054E-08 | 0,207E-04 ± 0,049E-04 | 0,627E-04 ± 0,276E-04 | 0,004E-04 ± 0,001E-04 | 0,452E-04 ± 0,185E-04 |
|  |  | Females | 0,010E-04 ± 0,006E-04 | NA | 4,467E-08 ± 4,501E-08 | 0,023E-04 ± 0,013E-04 | 0,954E-04 ± 1,092E-04 | 0,003E+00 ± 4,742E-04 | 0,013E-04 ± 0,016E-04 | 5,758E-08 ± 2,575E-08 | 0,005E-04 ± 0,012E-04 | 8,736E-08 ± 4,459E-08 | 0,228E-04 ± 0,053E-04 | 0,689E-04 ± 0,303E-04 | 0,004E-04 ± 0,002E-04 | 0,497E-04 ± 0,204E-04 |
|  | Children | Males | 0,007E-04 ± 0,005E-04 | NA | 3,102E-08 ± 3,126E-08 | 0,016E-04 ± 0,009E-04 | 0,662E-04 ± 0,759E-04 | 0,002E+00 ± 3,293E-04 | 0,009E-04 ± 0,011E-04 | 3,998E-08 ± 1,788E-08 | 0,003E-04 ± 0,008E-04 | 6,067E-08 ± 3,097E-08 | 0,158E-04 ± 0,037E-04 | 0,479E-04 ± 0,211E-04 | 0,003E-04 ± 0,001E-04 | 0,345E-04 ± 0,142E-04 |
|  |  | Females | 0,008E-04 ± 0,005E-04 | NA | 3,384E-08 ± 3,410E-08 | 0,017E-04 ± 0,009E-04 | 0,722E-04 ± 0,828E-04 | 0,003E+00 ± 3,592E-04 | 0,010E-04 ± 0,012E-04 | 4,362E-08 ± 1,951E-08 | 0,004E-04 ± 0,009E-04 | 6,618E-08 ± 3,378E-08 | 0,172E-04 ± 0,040E-04 | 0,522E-04 ± 0,230E-04 | 0,003E-04 ± 0,001E-04 | 0,376E-04 ± 0,154E-04 |
|  | Adults | Males | 0,005E-04 ± 0,003E-04 | NA | 2,127E-08 ± 2,143E-08 | 0,011E-04 ± 0,006E-04 | 0,454E-04 ± 0,520E-04 | 0,002E+00 ± 2,258E-04 | 0,006E-04 ± 0,008E-04 | 2,742E-08 ± 1,226E-08 | 0,002E-04 ± 0,006E-04 | 4,160E-08 ± 2,124E-08 | 0,108E-04 ± 0,025E-04 | 0,328E-04 ± 0,144E-04 | 0,002E-04 ± 7,453E-08 | 0,237E-04 ± 0,097E-04 |
|  |  | Females | 0,005E-04 ± 0,003E-04 | NA | 2,233E-08 ± 2,251E-08 | 0,011E-04 ± 0,006E-04 | 0,477E-04 ± 0,546E-04 | 0,002E+00 ± 2,371E-04 | 0,007E-04 ± 0,008E-04 | 2,879E-08 ± 1,288E-08 | 0,002E-04 ± 0,006E-04 | 4,368E-08 ± 2,230E-08 | 0,114E-04 ± 0,027E-04 | 0,345E-04 ± 0,152E-04 | 0,002E-04 ± 7,826E-08 | 0,248E-04 ± 0,102E-04 |
| **EWI Dry Season** | Babies | Males | 0,004E-04 ± 0,002E-04 | 6,334E-08 ± 3,536E-08 | 6,421E-08 ± 3,915E-08 | 0,025E-04 ± 0,013E-04 | 0,917E-04 ± 0,465E-04 | 0,003E+00 ± 5,134E-04 | 0,025E-04 ± 0,018E-04 | 0,003E-04 ± 0,007E-04 | 0,007E-04 ± 0,012E-04 | 0,002E-04 ± 0,002E-04 | 0,175E-04 ± 0,032E-04 | 0,606E-04 ± 0,156E-04 | 0,002E-04 ± 5,122E-08 | 0,439E-04 ± 0,086E-04 |
|  |  | Females | 0,004E-04 ± 0,002E-04 | 6,968E-08 ± 3,890E-08 | 7,063E-08 ± 4,306E-08 | 0,028E-04 ± 0,014E-04 | 1,008E-04 ± 0,512E-04 | 0,003E+00 ± 5,647E-04 | 0,027E-04 ± 0,020E-04 | 0,004E-04 ± 0,008E-04 | 0,007E-04 ± 0,013E-04 | 0,002E-04 ± 0,002E-04 | 0,192E-04 ± 0,035E-04 | 0,667E-04 ± 0,171E-04 | 0,003E-04 ± 5,635E-08 | 0,483E-04 ± 0,095E-04 |
|  | Children | Males | 0,003E-04 ± 0,001E-04 | 4,839E-08 ± 2,701E-08 | 4,905E-08 ± 2,990E-08 | 0,019E-04 ± 0,010E-04 | 0,700E-04 ± 0,355E-04 | 0,002E+00 ± 3,922E-04 | 0,019E-04 ± 0,014E-04 | 0,002E-04 ± 0,006E-04 | 0,005E-04 ± 0,009E-04 | 0,001E-04 ± 0,001E-04 | 0,133E-04 ± 0,024E-04 | 0,463E-04 ± 0,119E-04 | 0,002E-04 ± 3,913E-08 | 0,335E-04 ± 0,066E-04 |
|  |  | Females | 0,003E-04 ± 0,001E-04 | 5,279E-08 ± 2,947E-08 | 5,351E-08 ± 3,262E-08 | 0,021E-04 ± 0,011E-04 | 0,764E-04 ± 0,388E-04 | 0,003E+00 ± 4,278E-04 | 0,021E-04 ± 0,015E-04 | 0,003E-04 ± 0,006E-04 | 0,006E-04 ± 0,010E-04 | 0,001E-04 ± 0,002E-04 | 0,145E-04 ± 0,026E-04 | 0,505E-04 ± 0,130E-04 | 0,002E-04 ± 4,269E-08 | 0,366E-04 ± 0,072E-04 |
|  | Adults | Males | 0,002E-04 ± 9,153E-08 | 3,318E-08 ± 1,852E-08 | 3,363E-08 ± 2,051E-08 | 0,013E-04 ± 0,007E-04 | 0,480E-04 ± 0,244E-04 | 0,002E+00 ± 2,689E-04 | 0,013E-04 ± 0,010E-04 | 0,002E-04 ± 0,004E-04 | 0,004E-04 ± 0,006E-04 | 8,400E-08 ± 9,856E-08 | 0,091E-04 ± 0,017E-04 | 0,318E-04 ± 0,082E-04 | 0,001E-04 ± 2,683E-08 | 0,230E-04 ± 0,045E-04 |
|  |  | Females | 0,002E-04 ± 9,611E-08 | 3,484E-08 ± 1,945E-08 | 3,531E-08 ± 2,153E-08 | 0,014E-04 ± 0,007E-04 | 0,504E-04 ± 0,256E-04 | 0,002E+00 ± 2,824E-04 | 0,014E-04 ± 0,010E-04 | 0,002E-04 ± 0,004E-04 | 0,004E-04 ± 0,006E-04 | 8,820E-08 ± 0,001E-04 | 0,096E-04 ± 0,017E-04 | 0,333E-04 ± 0,086E-04 | 0,001E-04 ± 2,817E-08 | 0,241E-04 ± 0,047E-04 |
| **EWI Rainy and Dry Season** | Babies | Males | 6.308E-04 ± 5.138E-04 | 6.334E-05 ± 3.536E-05 | 5.279E-05 ± 4.143E-05 | 2.306E-03 ± 1.216E-03 | 8.926E-02 ± 7.612E-02 | 3.097E+00 ± 4.756E-01 | 1.847E-03 ± 1.758E-03 | 1.699E-04 ± 4.785E-04 | 5.655E-04 ± 1.110E-03 | 1.227E-04 ± 1.451E-04 | 1.902E-02 ± 4.351E-03 | 6.161E-02 ± 2.204E-02 | 3.035E-04 ± 1.254E-04 | 4.451E-02 ± 1.419E-02 |
|  |  | Females | 6.939E-04 ± 5.652E-04 | 6.968E-05 ± 3.890E-05 | 5.807E-05 ± 4.557E-05 | 2.537E-03 ± 1.338E-03 | 9.819E-02 ± 8.374E-02 | 3.407E+00 ± 5.232E-01 | 2.031E-03 ± 1.934E-03 | 1.869E-04 ± 5.264E-04 | 6.221E-04 ± 1.221E-03 | 1.350E-04 ± 1.596E-04 | 2.093E-02 ± 4.786E-03 | 6.777E-02 ± 2.424E-02 | 3.338E-04 ± 1.380E-04 | 4.896E-02 ± 1.560E-02 |
|  | Children | Males | 4.819E-04 ± 3.925E-04 | 4.839E-05 ± 2.701E-05 | 4.032E-05 ± 3.165E-05 | 1.762E-03 ± 9.290E-04 | 6.818E-02 ± 5.815E-02 | 2.366E+00 ± 3.633E-01 | 1.411E-03 ± 1.343E-03 | 1.298E-04 ± 3.656E-04 | 4.320E-04 ± 8.478E-04 | 9.372E-05 ± 1.108E-04 | 1.453E-02 ± 3.323E-03 | 4.706E-02 ± 1.683E-02 | 2.318E-04 ± 9.580E-05 | 3.400E-02 ± 1.084E-02 |
|  |  | Females | 5.257E-04 ± 4.282E-04 | 5.279E-05 ± 2.947E-05 | 4.399E-05 ± 3.452E-05 | 1.922E-03 ± 1.014E-03 | 7.438E-02 ± 6.344E-02 | 2.581E+00 ± 3.964E-01 | 1.539E-03 ± 1.465E-03 | 1.416E-04 ± 3.988E-04 | 4.713E-04 ± 9.249E-04 | 1.022E-04 ± 1.209E-04 | 1.585E-02 ± 3.626E-03 | 5.134E-02 ± 1.836E-02 | 2.529E-04 ± 1.045E-04 | 3.709E-02 ± 1.182E-02 |
|  | Adults | Males | 3.304E-04 ± 2.692E-04 | 3.318E-05 ± 1.852E-05 | 2.765E-05 ± 2.170E-05 | 1.208E-03 ± 6.371E-04 | 4.676E-02 ± 3.987E-02 | 1.622E+00 ± 2.491E-01 | 9.673E-04 ± 9.208E-04 | 8.900E-05 ± 2.507E-04 | 2.962E-04 ± 5.813E-04 | 6.426E-05 ± 7.598E-05 | 9.965E-03 ± 2.279E-03 | 3.227E-02 ± 1.154E-02 | 1.590E-04 ± 6.569E-05 | 2.332E-02 ± 7.431E-03 |
|  |  | Females | 3.470E-04 ± 2.826E-04 | 3.484E-05 ± 1.945E-05 | 2.903E-05 ± 2.278E-05 | 1.268E-03 ± 6.689E-04 | 4.909E-02 ± 4.187E-02 | 1.703E+00 ± 2.616E-01 | 1.016E-03 ± 9.668E-04 | 9.345E-05 ± 2.632E-04 | 3.111E-04 ± 6.104E-04 | 6.748E-05 ± 7.978E-05 | 1.046E-02 ± 2.393E-03 | 3.389E-02 ± 1.212E-02 | 1.669E-04 ± 6.898E-05 | 2.448E-02 ± 7.802E-03 |
| * EFSA, (2006);FAO/ WHO, (1995);USEPA, (2000)- Cu: Babies (<1 mg kg⁻¹); Children (<3 mg kg⁻¹); Adults (<5 mg kg⁻¹); Zn: Babies (<7 mg kg⁻¹); Children (<22 mg kg⁻¹); Adults (<25 mg kg⁻¹) | | | | | | | | | | | | | | | | |

**Table S5:** Target cancer risk (TCR) calculated for arsenic (As), 1% and 10% from consumption of Oreochromis niloticus across age groups, consumption frequencies (including once per week), and dry, rainy, and mixed seasons. Carcinogenic risks are noted when values are > 1 × 10^− 4^ (Antoine et al., 2017; USEPA, 2012).

|  | **Group** | **Sex** | **As (1x)** | **As (2x)** | **As (3x)** | **As (4x)** | **As (5x)** | **As 1% (1x)** | **As 1% (2x)** | **As 1% (3x)** | **As 1% (4x)** | **As 1% (5x)** | **As 10% (1x)** | **As 10% (2x)** | **As 10% (3x)** | **As 10% (4x)** | **As 10% (5x)** |
| --- | --- | --- | --- | --- | --- | --- | --- | --- | --- | --- | --- | --- | --- | --- | --- | --- | --- |
| **TCR Rainy Season** | Babies | Males | 3.77213E-08 | 7.54427E-08 | 1.13164E-07 | 1.50885E-07 | 1.88607E-07 | 3.77213E-10 | 7.54427E-10 | 1.13164E-09 | 1.50885E-09 | 1.88607E-09 | 3.77213E-09 | 7.54427E-09 | 1.13164E-08 | 1.50885E-08 | 1.88607E-08 |
|  |  | Females | 4.14935E-08 | 8.29869E-08 | 1.2448E-07 | 1.65974E-07 | 2.07467E-07 | 4.14935E-10 | 8.29869E-10 | 1.2448E-09 | 1.65974E-09 | 2.07467E-09 | 4.14935E-09 | 8.29869E-09 | 1.2448E-08 | 1.65974E-08 | 2.07467E-08 |
|  | Children | Males | 2.26403E-08 | 4.52806E-08 | 6.79209E-08 | 9.05612E-08 | 1.13201E-07 | 2.26403E-10 | 4.52806E-10 | 6.79209E-10 | 9.05612E-10 | 1.13201E-09 | 2.26403E-09 | 4.52806E-09 | 6.79209E-09 | 9.05612E-09 | 1.13201E-08 |
|  |  | Females | 2.46985E-08 | 4.9397E-08 | 7.40955E-08 | 9.8794E-08 | 1.23492E-07 | 2.46985E-10 | 4.9397E-10 | 7.40955E-10 | 9.8794E-10 | 1.23492E-09 | 2.46985E-09 | 4.9397E-09 | 7.40955E-09 | 9.8794E-09 | 1.23492E-08 |
|  | Adults | Males | 1.49186E-08 | 2.98371E-08 | 4.47557E-08 | 5.96742E-08 | 7.45928E-08 | 1.49186E-10 | 2.98371E-10 | 4.47557E-10 | 5.96742E-10 | 7.45928E-10 | 1.49186E-09 | 2.98371E-09 | 4.47557E-09 | 5.96742E-09 | 7.45928E-09 |
|  |  | Females | 1.56645E-08 | 3.1329E-08 | 4.69934E-08 | 6.26579E-08 | 7.83224E-08 | 1.56645E-10 | 3.1329E-10 | 4.69934E-10 | 6.26579E-10 | 7.83224E-10 | 1.56645E-09 | 3.1329E-09 | 4.69934E-09 | 6.26579E-09 | 7.83224E-09 |
| **TCR Dry Season** | Babies | Males | 1.43864E-08 | 2.87729E-08 | 4.31593E-08 | 5.75458E-08 | 7.19322E-08 | 1.43864E-10 | 2.87729E-10 | 4.31593E-10 | 5.75458E-10 | 7.19322E-10 | 1.43864E-09 | 2.87729E-09 | 4.31593E-09 | 5.75458E-09 | 7.19322E-09 |
|  |  | Females | 1.58251E-08 | 3.16502E-08 | 4.74753E-08 | 6.33004E-08 | 7.91255E-08 | 1.58251E-10 | 3.16502E-10 | 4.74753E-10 | 6.33004E-10 | 7.91255E-10 | 1.58251E-09 | 3.16502E-09 | 4.74753E-09 | 6.33004E-09 | 7.91255E-09 |
|  | Children | Males | 8.63472E-09 | 1.72694E-08 | 2.59042E-08 | 3.45389E-08 | 4.31736E-08 | 8.63472E-11 | 1.72694E-10 | 2.59042E-10 | 3.45389E-10 | 4.31736E-10 | 8.63472E-10 | 1.72694E-09 | 2.59042E-09 | 3.45389E-09 | 4.31736E-09 |
|  |  | Females | 9.4197E-09 | 1.88394E-08 | 2.82591E-08 | 3.76788E-08 | 4.70985E-08 | 9.4197E-11 | 1.88394E-10 | 2.82591E-10 | 3.76788E-10 | 4.70985E-10 | 9.4197E-10 | 1.88394E-09 | 2.82591E-09 | 3.76788E-09 | 4.70985E-09 |
|  | Adults | Males | 5.68975E-09 | 1.13795E-08 | 1.70693E-08 | 2.2759E-08 | 2.84488E-08 | 5.68975E-11 | 1.13795E-10 | 1.70693E-10 | 2.2759E-10 | 2.84488E-10 | 5.68975E-10 | 1.13795E-09 | 1.70693E-09 | 2.2759E-09 | 2.84488E-09 |
|  |  | Females | 5.97424E-09 | 1.19485E-08 | 1.79227E-08 | 2.3897E-08 | 2.98712E-08 | 5.97424E-11 | 1.19485E-10 | 1.79227E-10 | 2.3897E-10 | 2.98712E-10 | 5.97424E-10 | 1.19485E-09 | 1.79227E-09 | 2.3897E-09 | 2.98712E-09 |
| **TCR Rainy and Dry Season** | Babies | Males | 2.56775E-08 | 5.13551E-08 | 7.70326E-08 | 1.0271E-07 | 1.28388E-07 | 2.56775E-10 | 5.13551E-10 | 7.70326E-10 | 1.0271E-09 | 1.28388E-09 | 2.56775E-09 | 5.13551E-09 | 7.70326E-09 | 1.0271E-08 | 1.28388E-08 |
|  |  | Females | 2.82453E-08 | 5.64906E-08 | 8.47358E-08 | 1.12981E-07 | 1.41226E-07 | 2.82453E-10 | 5.64906E-10 | 8.47358E-10 | 1.12981E-09 | 1.41226E-09 | 2.82453E-09 | 5.64906E-09 | 8.47358E-09 | 1.12981E-08 | 1.41226E-08 |
|  | Children | Males | 1.54116E-08 | 3.08232E-08 | 4.62348E-08 | 6.16464E-08 | 7.70581E-08 | 1.54116E-10 | 3.08232E-10 | 4.62348E-10 | 6.16464E-10 | 7.70581E-10 | 1.54116E-09 | 3.08232E-09 | 4.62348E-09 | 6.16464E-09 | 7.70581E-09 |
|  |  | Females | 1.68127E-08 | 3.36253E-08 | 5.0438E-08 | 6.72507E-08 | 8.40633E-08 | 1.68127E-10 | 3.36253E-10 | 5.0438E-10 | 6.72507E-10 | 8.40633E-10 | 1.68127E-09 | 3.36253E-09 | 5.0438E-09 | 6.72507E-09 | 8.40633E-09 |
|  | Adults | Males | 1.01553E-08 | 2.03106E-08 | 3.04659E-08 | 4.06212E-08 | 5.07765E-08 | 1.01553E-10 | 2.03106E-10 | 3.04659E-10 | 4.06212E-10 | 5.07765E-10 | 1.01553E-09 | 2.03106E-09 | 3.04659E-09 | 4.06212E-09 | 5.07765E-09 |
|  |  | Females | 1.06631E-08 | 2.13261E-08 | 3.19892E-08 | 4.26523E-08 | 5.33153E-08 | 1.06631E-10 | 2.13261E-10 | 3.19892E-10 | 4.26523E-10 | 5.33153E-10 | 1.06631E-09 | 2.13261E-09 | 3.19892E-09 | 4.26523E-09 | 5.33153E-09 |
|  | | | | | | | | | | | | | | | | |  |

**Table S6:** Target cancer risk (TCR) calculated for cadmium (Cd) and lead (Pb) from consumption of Oreochromis niloticus across age groups, consumption frequencies (including once per week), and dry, rainy, and mixed seasons. Carcinogenic risks are noted when values are > 1 × 10^− 4^ (Antoine et al., 2017; USEPA, 2012).

|  | **Group** | **Sex** | **Cd (1x)** | **Cd (2x)** | **Cd (3x)** | **Cd (4x)** | **Cd (5x)** | **Pb (1x)** | **Pb (2x)** | **Pb (3x)** | **Pb (4x)** | **Pb (5x)** |  |  |
| --- | --- | --- | --- | --- | --- | --- | --- | --- | --- | --- | --- | --- | --- | --- |
| **TCR Rainy Season** | Babies | Males | NA | NA | NA | NA | NA | 1.83183E-11 | 3.66365E-11 | 5.49548E-11 | 7.32731E-11 | 9.15913E-11 |  |  |
|  |  | Females | NA | NA | NA | NA | NA | 2.01501E-11 | 4.03002E-11 | 6.04503E-11 | 8.06004E-11 | 1.0075E-10 |  |  |
|  | Children | Males | NA | NA | NA | NA | NA | 1.09946E-11 | 2.19892E-11 | 3.29838E-11 | 4.39784E-11 | 5.4973E-11 |  |  |
|  |  | Females | NA | NA | NA | NA | NA | 1.19941E-11 | 2.39882E-11 | 3.59823E-11 | 4.79764E-11 | 5.99705E-11 |  |  |
|  | Adults | Males | NA | NA | NA | NA | NA | 7.24476E-12 | 1.44895E-11 | 2.17343E-11 | 2.8979E-11 | 3.62238E-11 |  |  |
|  |  | Females | NA | NA | NA | NA | NA | 7.607E-12 | 1.5214E-11 | 2.2821E-11 | 3.0428E-11 | 3.8035E-11 |  |  |
| **TCR Dry Season** | Babies | Males | 1.71895E-12 | 3.43789E-12 | 5.15684E-12 | 6.87578E-12 | 8.59473E-12 | 3.69903E-11 | 7.39806E-11 | 1.10971E-10 | 1.47961E-10 | 1.84952E-10 |  |  |
|  |  | Females | 1.89084E-12 | 3.78168E-12 | 5.67252E-12 | 7.56336E-12 | 9.4542E-12 | 4.06894E-11 | 8.13787E-11 | 1.22068E-10 | 1.62757E-10 | 2.03447E-10 |  |  |
|  | Children | Males | 1.03171E-12 | 2.06342E-12 | 3.09513E-12 | 4.12683E-12 | 5.15854E-12 | 2.22015E-11 | 4.44031E-11 | 6.66046E-11 | 8.88061E-11 | 1.11008E-10 |  |  |
|  |  | Females | 1.1255E-12 | 2.251E-12 | 3.3765E-12 | 4.502E-12 | 5.6275E-12 | 2.42199E-11 | 4.84397E-11 | 7.26596E-11 | 9.68794E-11 | 1.21099E-10 |  |  |
|  | Adults | Males | 6.79832E-13 | 1.35966E-12 | 2.0395E-12 | 2.71933E-12 | 3.39916E-12 | 1.46294E-11 | 2.92589E-11 | 4.38883E-11 | 5.85178E-11 | 7.31472E-11 |  |  |
|  |  | Females | 7.13824E-13 | 1.42765E-12 | 2.14147E-12 | 2.8553E-12 | 3.56912E-12 | 1.53609E-11 | 3.07218E-11 | 4.60827E-11 | 6.14437E-11 | 7.68046E-11 |  |  |
| **TCR Rainy and Dry Season** | Babies | Males | 6.53199E-12 | 1.3064E-11 | 1.9596E-11 | 2.6128E-11 | 3.266E-11 | 2.82982E-11 | 5.65963E-11 | 8.48945E-11 | 1.13193E-10 | 1.41491E-10 |  |  |
|  |  | Females | 7.18519E-12 | 1.43704E-11 | 2.15556E-11 | 2.87408E-11 | 3.5926E-11 | 3.1128E-11 | 6.22559E-11 | 9.33839E-11 | 1.24512E-10 | 1.5564E-10 |  |  |
|  | Children | Males | 3.92049E-12 | 7.84098E-12 | 1.17615E-11 | 1.5682E-11 | 1.96025E-11 | 1.69845E-11 | 3.3969E-11 | 5.09535E-11 | 6.7938E-11 | 8.49225E-11 |  |  |
|  |  | Females | 4.2769E-12 | 8.5538E-12 | 1.28307E-11 | 1.71076E-11 | 2.13845E-11 | 1.85286E-11 | 3.70571E-11 | 5.55857E-11 | 7.41142E-11 | 9.26428E-11 |  |  |
|  | Adults | Males | 2.58336E-12 | 5.16673E-12 | 7.75009E-12 | 1.03335E-11 | 1.29168E-11 | 1.11917E-11 | 2.23835E-11 | 3.35752E-11 | 4.4767E-11 | 5.59587E-11 |  |  |
|  |  | Females | 2.71253E-12 | 5.42506E-12 | 8.13759E-12 | 1.08501E-11 | 1.35627E-11 | 1.17513E-11 | 2.35027E-11 | 3.5254E-11 | 4.70053E-11 | 5.87567E-11 |  |  |

**Table S7:** Target Hazard Quotient (THQ) for selected metals and metalloids by age group, sex, and consumption frequency concerning Oreochromis niloticus specimens sampled from Jacarepaguá lagoon complex (JLC), Rio de Janeiro, Southeastern Brazil. Values above 1, indicative of human health risks, are indicated in bold.

|  | **Group** | **Sex** | **As** | **As (1%)** | **As (10%)** | **Cu** | **Ni** | **V** | **Zn** | **HI** | **Frequency** |  |  |
| --- | --- | --- | --- | --- | --- | --- | --- | --- | --- | --- | --- | --- | --- |
| **THQ Rainy Season** | Babies | Males | 0.08383 | 0.00084 | 0.00838 | 0.01405 | 0.00107 | 0.00045 | 0.00409 | 0.10416 | 1x |  |  |
|  |  | Females | 0.09221 | 0.00092 | 0.00922 | 0.01545 | 0.00118 | 0.00124 | 0.00450 | 0.11458 |  |  |  |
|  | Children | Males | 0.05031 | 0.00050 | 0.00503 | 0.00843 | 0.00064 | 0.00113 | 0.00245 | 0.06252 |  |  |  |
|  |  | Females | 0.05489 | 0.00055 | 0.00549 | 0.00920 | 0.00070 | 0.00074 | 0.00268 | 0.06820 |  |  |  |
|  | Adults | Males | 0.03315 | 0.00033 | 0.00332 | 0.00556 | 0.00042 | 0.00068 | 0.00162 | 0.04119 |  |  |  |
|  |  | Females | 0.03481 | 0.00035 | 0.00348 | 0.00583 | 0.00045 | 0.00047 | 0.00170 | 0.04325 |  |  |  |
| **THQ Dry Season** | Babies | Males | 0.03197 | 0.00032 | 0.00320 | 0.01714 | 0.00165 | 0.00071 | 0.00397 | 0.05545 | 1x |  |  |
|  |  | Females | 0.03516 | 0.00035 | 0.00352 | 0.01886 | 0.00181 | 0.00079 | 0.00437 | 0.06099 |  |  |  |
|  | Children | Males | 0.01918 | 0.00019 | 0.00192 | 0.01029 | 0.00099 | 0.00043 | 0.00238 | 0.03328 |  |  |  |
|  |  | Females | 0.02093 | 0.00021 | 0.00209 | 0.01122 | 0.00108 | 0.00047 | 0.00260 | 0.03630 |  |  |  |
|  | Adults | Males | 0.01264 | 0.00013 | 0.00126 | 0.00678 | 0.00065 | 0.00028 | 0.00157 | 0.02193 |  |  |  |
|  |  | Females | 0.01327 | 0.00013 | 0.00133 | 0.00712 | 0.00069 | 0.00030 | 0.00165 | 0.02303 |  |  |  |
| **THQ Rainy and Dry Season** | Babies | Males | 0.05706 | 0.00057 | 0.00570 | 0.01564 | 0.00137 | 0.00091 | 0.00402 | 0.07902 | 1x |  |  |
|  |  | Females | 0.06276 | 0.00062 | 0.00627 | 0.01720 | 0.00150 | 0.00100 | 0.00442 | 0.08692 |  |  |  |
|  | Children | Males | 0.03424 | 0.00034 | 0.00342 | 0.00939 | 0.00082 | 0.00054 | 0.00241 | 0.04743 |  |  |  |
|  |  | Females | 0.03736 | 0.00037 | 0.00373 | 0.01024 | 0.00089 | 0.00059 | 0.00263 | 0.05174 |  |  |  |
|  | Adults | Males | 0.02256 | 0.00022 | 0.00225 | 0.00618 | 0.00054 | 0.00036 | 0.00159 | 0.03125 |  |  |  |
|  |  | Females | 0.02369 | 0.00023 | 0.00236 | 0.00649 | 0.00056 | 0.00037 | 0.00167 | 0.03281 |  |  |  |

**Table S8:** Target Hazard Quotient (THQ) for selected metals and metalloids by age group, sex, and consumption frequency concerning Oreochromis niloticus specimens sampled from Jacarepaguá lagoon complex (JLC), Rio de Janeiro, Southeastern Brazil. Values above 1, indicative of human health risks, are indicated in bold.

|  | **Group** | **Sex** | **As** | **As (1%)** | **As (10%)** | **Cu** | **Ni** | **V** | **Zn** | **HI** | **Frequency** |  |  |
| --- | --- | --- | --- | --- | --- | --- | --- | --- | --- | --- | --- | --- | --- |
| **THQ Rainy Season** | Babies | Males | 0.16765 | 0.00168 | 0.01677 | 0.02810 | 0.00214 | 0.00226 | 0.00817 | 0.20832 | 2x |  |  |
|  |  | Females | 0.18442 | 0.00184 | 0.01844 | 0.03091 | 0.00236 | 0.00248 | 0.00899 | 0.22915 |  |  |  |
|  | Children | Males | 0.10062 | 0.00101 | 0.01006 | 0.01686 | 0.00129 | 0.00135 | 0.00491 | 0.12503 |  |  |  |
|  |  | Females | 0.10977 | 0.00110 | 0.01098 | 0.01840 | 0.00140 | 0.00148 | 0.00535 | 0.13640 |  |  |  |
|  | Adults | Males | 0.06630 | 0.00066 | 0.00663 | 0.01111 | 0.00085 | 0.00089 | 0.00323 | 0.08239 |  |  |  |
|  |  | Females | 0.06962 | 0.00070 | 0.00696 | 0.01167 | 0.00089 | 0.00094 | 0.00339 | 0.08651 |  |  |  |
| **THQ Dry Season** | Babies | Males | 0.06394 | 0.00064 | 0.00639 | 0.03428 | 0.00330 | 0.00143 | 0.00794 | 0.11089 | 2x |  |  |
|  |  | Females | 0.07033 | 0.00070 | 0.00703 | 0.03771 | 0.00363 | 0.00157 | 0.00873 | 0.12198 |  |  |  |
|  | Children | Males | 0.03838 | 0.00038 | 0.00384 | 0.02058 | 0.00198 | 0.00086 | 0.00477 | 0.06656 |  |  |  |
|  |  | Females | 0.04187 | 0.00042 | 0.00419 | 0.02245 | 0.00216 | 0.00094 | 0.00520 | 0.07261 |  |  |  |
|  | Adults | Males | 0.02529 | 0.00025 | 0.00253 | 0.01356 | 0.00131 | 0.00057 | 0.00314 | 0.04386 |  |  |  |
|  |  | Females | 0.02655 | 0.00027 | 0.00266 | 0.01424 | 0.00137 | 0.00059 | 0.00330 | 0.04605 |  |  |  |
| **THQ Rainy and Dry Season** | Babies | Males | 0.11412 | 0.00114 | 0.01141 | 0.03128 | 0.00274 | 0.00183 | 0.00805 | 0.15804 | 2x |  |  |
|  |  | Females | 0.12553 | 0.00125 | 0.01255 | 0.03441 | 0.00301 | 0.00201 | 0.00885 | 0.17384 |  |  |  |
|  | Children | Males | 0.06849 | 0.00068 | 0.00684 | 0.01878 | 0.00164 | 0.00109 | 0.00483 | 0.09485 |  |  |  |
|  |  | Females | 0.07472 | 0.00074 | 0.00747 | 0.02048 | 0.00179 | 0.00119 | 0.00527 | 0.10348 |  |  |  |
|  | Adults | Males | 0.04513 | 0.00045 | 0.00451 | 0.01237 | 0.00108 | 0.00072 | 0.00318 | 0.06250 |  |  |  |
|  |  | Females | 0.04739 | 0.00047 | 0.00473 | 0.01299 | 0.00113 | 0.00075 | 0.00334 | 0.06563 |  |  |  |

**Table S9:** Target Hazard Quotient (THQ) for selected metals and metalloids by age group, sex, and consumption frequency concerning Oreochromis niloticus specimens sampled from Jacarepaguá lagoon complex (JLC), Rio de Janeiro, Southeastern Brazil. Values above 1, indicative of human health risks, are indicated in bold.

|  | **Group** | **Sex** | **As** | **As (1%)** | **As (10%)** | **Cu** | **Ni** | **V** | **Zn** | **HI** | **Frequency** |  |  |
| --- | --- | --- | --- | --- | --- | --- | --- | --- | --- | --- | --- | --- | --- |
| **THQ Rainy Season** | Babies | Males | 0.25148 | 0.00251 | 0.02515 | 0.04214 | 0.00322 | 0.00339 | 0.01226 | 0.31248 | 3x |  |  |
|  |  | Females | 0.27662 | 0.00277 | 0.02766 | 0.04636 | 0.00354 | 0.00372 | 0.01349 | 0.34373 |  |  |  |
|  | Children | Males | 0.15094 | 0.00151 | 0.01509 | 0.02529 | 0.00193 | 0.00203 | 0.00736 | 0.18755 |  |  |  |
|  |  | Females | 0.16466 | 0.00165 | 0.01647 | 0.02759 | 0.00211 | 0.00222 | 0.00803 | 0.20460 |  |  |  |
|  | Adults | Males | 0.09946 | 0.00099 | 0.00995 | 0.01667 | 0.00127 | 0.00134 | 0.00485 | 0.12358 |  |  |  |
|  |  | Females | 0.10443 | 0.00104 | 0.01044 | 0.01750 | 0.00134 | 0.00141 | 0.00509 | 0.12976 |  |  |  |
| **THQ Dry Season** | Babies | Males | 0.09591 | 0.00096 | 0.00959 | 0.05143 | 0.00495 | 0.00214 | 0.01310 | 0.16634 | 3x |  |  |
|  |  | Females | 0.10550 | 0.00106 | 0.01055 | 0.05657 | 0.00544 | 0.00236 | 0.01191 | 0.18297 |  |  |  |
|  | Children | Males | 0.05756 | 0.00058 | 0.00576 | 0.03087 | 0.00297 | 0.00129 | 0.00780 | 0.09984 |  |  |  |
|  |  | Females | 0.06280 | 0.00063 | 0.00628 | 0.03367 | 0.00324 | 0.00140 | 0.00715 | 0.10891 |  |  |  |
|  | Adults | Males | 0.03793 | 0.00038 | 0.00379 | 0.02034 | 0.00196 | 0.00085 | 0.00495 | 0.06579 |  |  |  |
|  |  | Females | 0.03983 | 0.00040 | 0.00398 | 0.02136 | 0.00206 | 0.00089 | 0.00471 | 0.06908 |  |  |  |
| **THQ Rainy and Dry Season** | Babies | Males | 0.17118 | 0.00171 | 0.01711 | 0.04693 | 0.00411 | 0.00274 | 0.01207 | 0.23705 | 3x |  |  |
|  |  | Females | 0.18830 | 0.00188 | 0.01883 | 0.05162 | 0.00452 | 0.00301 | 0.01328 | 0.26076 |  |  |  |
|  | Children | Males | 0.10274 | 0.00102 | 0.01027 | 0.02817 | 0.00246 | 0.00164 | 0.00724 | 0.14228 |  |  |  |
|  |  | Females | 0.11208 | 0.00112 | 0.01120 | 0.03073 | 0.00269 | 0.00179 | 0.00790 | 0.15521 |  |  |  |
|  | Adults | Males | 0.06770 | 0.00067 | 0.00677 | 0.01856 | 0.00162 | 0.00108 | 0.00477 | 0.09375 |  |  |  |
|  |  | Females | 0.07108 | 0.00071 | 0.00710 | 0.01949 | 0.00170 | 0.00113 | 0.00501 | 0.09844 |  |  |  |

**Table S10:** Target Hazard Quotient (THQ) for selected metals and metalloids by age group, sex, and consumption frequency concerning Oreochromis niloticus specimens sampled from Jacarepaguá lagoon complex (JLC), Rio de Janeiro, Southeastern Brazil. Values above 1, indicative of human health risks, are indicated in bold.

|  | **Group** | **Sex** | **As** | **As (1%)** | **As (10%)** | **Cu** | **Ni** | **V** | **Zn** | **HI** | **Frequency** |  |  |
| --- | --- | --- | --- | --- | --- | --- | --- | --- | --- | --- | --- | --- | --- |
| **THQ Rainy Season** | Babies | Males | 0.33530 | 0.00335 | 0.03353 | 0.05619 | 0.00429 | 0.00451 | 0.01635 | 0.41664 | 4x |  |  |
|  |  | Females | 0.36883 | 0.00369 | 0.03688 | 0.06181 | 0.00472 | 0.00496 | 0.01798 | 0.45830 |  |  |  |
|  | Children | Males | 0.20125 | 0.00201 | 0.02012 | 0.03373 | 0.00257 | 0.00271 | 0.00981 | 0.25007 |  |  |  |
|  |  | Females | 0.21954 | 0.00220 | 0.02195 | 0.03679 | 0.00281 | 0.00296 | 0.01070 | 0.27280 |  |  |  |
|  | Adults | Males | 0.13261 | 0.00133 | 0.01326 | 0.02222 | 0.00170 | 0.00179 | 0.00647 | 0.16478 |  |  |  |
|  |  | Females | 0.13924 | 0.00139 | 0.01392 | 0.02333 | 0.00178 | 0.00187 | 0.00679 | 0.17302 |  |  |  |
| **THQ Dry Season** | Babies | Males | 0.12788 | 0.00128 | 0.01279 | 0.06857 | 0.00660 | 0.00286 | 0.01588 | 0.22179 | 4x |  |  |
|  |  | Females | 0.14067 | 0.00141 | 0.01407 | 0.07542 | 0.00726 | 0.00315 | 0.01747 | 0.24396 |  |  |  |
|  | Children | Males | 0.07675 | 0.00077 | 0.00768 | 0.04115 | 0.00396 | 0.00172 | 0.00953 | 0.13312 |  |  |  |
|  |  | Females | 0.08373 | 0.00084 | 0.00837 | 0.04490 | 0.00432 | 0.00187 | 0.01040 | 0.14522 |  |  |  |
|  | Adults | Males | 0.05058 | 0.00051 | 0.00506 | 0.02712 | 0.00261 | 0.00113 | 0.00628 | 0.08771 |  |  |  |
|  |  | Females | 0.05310 | 0.00053 | 0.00531 | 0.02847 | 0.00274 | 0.00119 | 0.00659 | 0.09210 |  |  |  |
| **THQ Rainy and Dry Season** | Babies | Males | 0.22824 | 0.00228 | 0.02282 | 0.06257 | 0.00548 | 0.00366 | 0.01610 | 0.31607 | 4x |  |  |
|  |  | Females | 0.25106 | 0.00251 | 0.02510 | 0.06883 | 0.00602 | 0.00402 | 0.01771 | 0.34768 |  |  |  |
|  | Children | Males | 0.13699 | 0.00136 | 0.01369 | 0.03756 | 0.00328 | 0.00219 | 0.00966 | 0.18970 |  |  |  |
|  |  | Females | 0.14944 | 0.00149 | 0.01494 | 0.04097 | 0.00358 | 0.00239 | 0.01054 | 0.20695 |  |  |  |
|  | Adults | Males | 0.09026 | 0.00090 | 0.00902 | 0.02474 | 0.00216 | 0.00144 | 0.00636 | 0.12500 |  |  |  |
|  |  | Females | 0.09478 | 0.00094 | 0.00947 | 0.02598 | 0.00227 | 0.00151 | 0.00668 | 0.13125 |  |  |  |

**Table S11:** Target Hazard Quotient (THQ) for selected metals and metalloids by age group, sex, and consumption frequency concerning Oreochromis niloticus specimens sampled from Jacarepaguá lagoon complex (JLC), Rio de Janeiro, Southeastern Brazil. Values above 1, indicative of human health risks, are indicated in bold.

|  | **Group** | **Sex** | **As** | **As (1%)** | **As (10%)** | **Cu** | **Ni** | **V** | **Zn** | **HI** | **Frequency** |  |  |
| --- | --- | --- | --- | --- | --- | --- | --- | --- | --- | --- | --- | --- | --- |
| **THQ Rainy Season** | Babies | Males | 0.41913 | 0.00419 | 0.04191 | 0.07024 | 0.00536 | 0.00564 | 0.02043 | 0.52080 | 5x |  |  |
|  |  | Females | 0.46104 | 0.00461 | 0.04610 | 0.07726 | 0.00590 | 0.00621 | 0.02248 | 0.57288 |  |  |  |
|  | Children | Males | 0.25156 | 0.00252 | 0.02516 | 0.04216 | 0.00322 | 0.00339 | 0.01226 | 0.31258 |  |  |  |
|  |  | Females | 0.27443 | 0.00274 | 0.02744 | 0.04599 | 0.00351 | 0.00369 | 0.01338 | 0.34100 |  |  |  |
|  | Adults | Males | 0.16576 | 0.00166 | 0.01658 | 0.02778 | 0.00212 | 0.00223 | 0.00808 | 0.20597 |  |  |  |
|  |  | Females | 0.17405 | 0.00174 | 0.01740 | 0.02917 | 0.00223 | 0.00234 | 0.00849 | 0.21627 |  |  |  |
| **THQ Dry Season** | Babies | Males | 0.15985 | 0.00160 | 0.01598 | 0.08571 | 0.00825 | 0.00357 | 0.01985 | 0.27723 | 5x |  |  |
|  |  | Females | 0.17583 | 0.00176 | 0.01758 | 0.09428 | 0.00907 | 0.00393 | 0.02183 | 0.30495 |  |  |  |
|  | Children | Males | 0.09594 | 0.00096 | 0.00959 | 0.05144 | 0.00495 | 0.00215 | 0.01191 | 0.16639 |  |  |  |
|  |  | Females | 0.10466 | 0.00105 | 0.01047 | 0.05612 | 0.00540 | 0.00234 | 0.01300 | 0.18152 |  |  |  |
|  | Adults | Males | 0.06322 | 0.00063 | 0.00632 | 0.03390 | 0.00326 | 0.00141 | 0.00785 | 0.10964 |  |  |  |
|  |  | Females | 0.06638 | 0.00066 | 0.00664 | 0.03559 | 0.00343 | 0.00148 | 0.00824 | 0.11513 |  |  |  |
| **THQ Rainy and Dry Season** | Babies | Males | 0.28530 | 0.00285 | 0.02853 | 0.07822 | 0.00685 | 0.00457 | 0.02013 | 0.39509 | 5x |  |  |
|  |  | Females | 0.31383 | 0.00313 | 0.03138 | 0.08604 | 0.00753 | 0.00503 | 0.02214 | 0.43460 |  |  |  |
|  | Children | Males | 0.17124 | 0.00171 | 0.01712 | 0.04695 | 0.00411 | 0.00274 | 0.01208 | 0.23713 |  |  |  |
|  |  | Females | 0.18680 | 0.00186 | 0.01868 | 0.05121 | 0.00448 | 0.00299 | 0.01318 | 0.25869 |  |  |  |
|  | Adults | Males | 0.11283 | 0.00112 | 0.01128 | 0.03093 | 0.00270 | 0.00180 | 0.00796 | 0.15625 |  |  |  |
|  |  | Females | 0.11847 | 0.00118 | 0.01184 | 0.03248 | 0.00284 | 0.00189 | 0.00835 | 0.16406 |  |  |  |

**Table S12:** Loading Values of Principal Component Analysis (PCA) of biomarker responses in Nile tilapia.

|  | PC1 | PC2 |
| --- | --- | --- |
| SOD | 0.139 | 0.444 |
| GST | 0.538 | -0.228 |
| LPO | 0.460 | 0.147 |
| PTC | 0.205 | 0.641 |
| Vtg_Female | 0.549 | 0.120 |
| Vtg_Male | 0.370 | -0.551 |

**Table S13:** Spearman correlations between antioxidant biomarkers, oxidative damage and vitellogenin in Nile tilapia (Oreochromis niloticus) from the Jacarepaguá Lagoon Complex (JLC).

|  | SOD | GST | GSH | TAC | MT | LPO | PTC | Vtg_Female | Vtg_Male |
| --- | --- | --- | --- | --- | --- | --- | --- | --- | --- |
| SOD | 1 |  |  |  |  |  |  |  |  |
| GST | 0.22 | 1 |  |  |  |  |  |  |  |
| GSH | 0.12 | -0.02 | 1 |  |  |  |  |  |  |
| TAC | -0.05 | -0.17 | 0.39* | 1 |  |  |  |  |  |
| MT | 0.18 | 0.10 | -0.24 | 0.22 | 1 |  |  |  |  |
| LPO | 0.01 | 0.54* | 0.02 | 0.06 | 0.27 | 1 |  |  |  |
| PTC | 0.36 | 0.38* | 0.22 | -0.16 | 0.15 | 0.35 | 1 |  |  |
| Vtg_Female | 0.005 | 0.26 | -0.006 | 0.06 | 0.03 | 0.14 | 0.24 | 1 |  |
| Vtg_Male | -0.05 | 0.11 | -0.30 | 0.05 | -0.23 | -0.001 | -0.33 | 0.15 | 1 |
| * Significant correlation (p < 0.05). | | | | | | | | | |
